# Effective macropore diffusivity of carbon dioxide on binderless pellets of Y-type zeolites

**DOI:** 10.1007/s10450-025-00599-3

**Published:** 2025-01-31

**Authors:** Hassan Azzan, Killian Gmyrek, David Danaci, Ashwin Kumar Rajagopalan, Camille Petit, Ronny Pini

**Affiliations:** 1 Department of Chemical Engineering, Imperial College London, London, SW7 2AZ UK; 2 The Sargent Centre for Process Systems Engineering, Imperial College London, London, SW7 2AZ UK; 3 I-X Centre for AI in Science, Imperial College London, London, W12 0BZ UK; 4 Department of Chemical Engineering, The University of Manchester, Manchester, M13 9PL UK

**Keywords:** Zero length column, Diffusion, Carbon Dioxide, Zeolites

## Abstract

The adsorption kinetics of carbon dioxide (CO_2_) in three cationic forms of binderless pellets of Y-types zeolites (H-Y, Na-Y, and TMA exchanged Na-Y) are studied using the zero-length column (ZLC) technique. The measurements were carried out at $288.15\,\textrm{K},298.15\,\textrm{K}$ and ${308.15}\,\textrm{K}$ using different flowrates and an initial CO_2_ partial pressure of ${0.10} \,\textrm{bar}$– conditions representative of post-combustion CO_2_ capture applications. The mass transport within the adsorbent pellets was described using a 1-D Fickian diffusion model accounting for intra- and inter-crystalline mass transport. For the latter, the parallel pore model formulation was used to explicitly account for the adsorbent’s macropore size distribution in estimating the volume-averaged diffusivity of the gas. Experiments carried out using different carrier gases, namely helium and nitrogen, were used (i) to determine that these systems are macropore diffusion limited and (ii) to simplify the parameter estimation to a single parameter - the macropore tortuosity. The latter ($\tau =1.3-2.5$) was in good agreement with independent measurements using MIP ($\tau \approx 1.7$). The associated diffusion coefficient, $D^\textrm{e}_\textrm{mac}$, was found to vary due to differences in the materials’ macropore size distributions and overall porosity. Upon combining the parallel pore model formulation with the temperature dependencies for the pore diffusivities derived from molecular theories of gases, we predict $D^\textrm{e}_\textrm{mac}\propto {T^b}$ with $b=[0.78-0.88]$ depending on the macropore size distribution. Notably, for the range of temperature tested in this study, $D^\textrm{e}_\textrm{mac}$ varies approximately linearly with temperature ($b\approx 1$)– in contrast to the commonly reported correlation of $b=1.75$, which may be more appropriate for systems where molecular diffusion dominates and Knudsen diffusion is negligible. The binderless pellets of Y-type zeolites studied exhibit generally higher values for the effective macropore diffusivity of CO_2_ compared to previously reported results on commercial FAU zeolites.

**Supplementary Information:**

The online version contains supplementary material available at 10.1007/s10450-025-00599-3.

## Introduction

Carbon dioxide (CO_2_) accounts for the majority of anthropogenic greenhouse gas emissions. Technologies for CO_2_ capture and removal are set to play an important role in mitigating the long-term effects of climate change [1]. The former entails abatement of emissions from point sources such as fossil-fuel-based power generation, manufacturing, and chemicals industries [2, 3], whilst the latter focuses on the net-removal of CO_2_ directly from the atmosphere [4]. Although amine-based absorption has reached high technological maturity [5], recent advancements in material science and computational process design have led to adsorption-based CO_2_ capture (and removal) gaining traction in terms of technical feasibility. This is in large part due to inherently lower energy demands and process versatility of adsorption-based separations [4, 6–9].

The exact properties of adsorbent materials that are favorable for each application vary, requiring the synergistic design of the process and adsorbent material. [10, 11] Two key properties that are particularly important in any application are the CO_2_ adsorption capacity at equilibrium and the adsorption kinetics. A review of the current literature on adsorbent characterization and development indicates that a greater emphasis has been traditionally placed on the former, with significantly less data being reported on the adsorption kinetics. Although the working capacity of a process is determined by the multi-component equilibrium capacities of the relevant gases in a mixture, incorrectly parameterized kinetics can result in large inaccuracies in predictions of key performance indicators (KPIs) for the given process [12, 13]. The relevant KPIs include the productivity (*i*.*e*., the amount of CO_2_ product separated per unit time and per unit volume of adsorbent) and the specific energy consumption, which can be obtained using high fidelity computational models that describe the unit operation [14]. Thus, accurate characterization of the adsorption kinetics, particularly for the heavy gas (*i*.*e*., CO_2_), is imperative to process design and techno-economic evaluation of adsorption-based CO_2_ capture and removal processes [15, 16].

The experimental characterization of adsorption kinetics encompasses a wide range of techniques, and in this work, we will focus on the zero-length column (ZLC) technique. The technique was first introduced to measure intra-crystalline diffusivity of strongly adsorbed species using a small amount of adsorbent material [17], typically in the order of 1 to 2 $\textrm{mg}$. The ZLC technique works on the principle that the use of a small amount of adsorbent reduces the adsorbent “bed” to a perfectly mixed cell (continuous stirred tank reactor, or CSTR) as the length of the column in the axial coordinate tends to zero. The main implication of this assumption is that in a simple desorption experiment with a carrier gas, the concentration of the adsorptive gas exiting the “column” can be assumed to be the same throughout the column. A ZLC experimental system can be used to extract kinetic parameters, such as intra-crystalline diffusivity using powder forms of the material, macropore diffusivity on shaped forms, as well as information on other transport mechanisms, such as surface barrier diffusional resistance [18]. Under certain experimental conditions, the technique can also be used to accurately extract adsorption equilibria by performing integration of the mass balance of the desorption curves. These details, along with other key contributions to the ZLC technique in terms of theory, experimental design, and interpretation since its inception, have been thoroughly discussed in the review by Brandani and Mangano [19].

To be used for practical separation applications, adsorbent materials need to be shaped into functional bodies (*e*.*g*. beads, pellets, etc.), and the adsorption kinetics on these forms need to be measured experimentally [20]. To our knowledge, the first study applying the ZLC technique to shaped pellets was carried out to study the diffusion of N_2_ and O_2_ in commercial 5A zeolites at a temperature of ${193}\,\textrm{K}$ [21]. Zeolites have continued to be extensively deployed for molecular gas separations and hold great potential as effective adsorbents for CO_2_ capture applications, too [22]. Kinetics of CO_2_ on shaped zeolites have been measured at near ambient temperatures on zeolite 13X using the ZLC technique [23–25]. Brandani *et al*. [26] also reported diffusion of p-xylene in commercial Y-zeolite pellets. These results unequivocally show that the overall mass transport rate of CO_2_ in shaped zeolite pellets is controlled by resistance to mass transfer in the macropores. In our previous work, we presented unary equilibrium data for crystalline Y-zeolites, namely, zeolite H-Y, Na-Y, and TMA exchanged Na-Y which were identified as promising materials for pre- and post-combustion CO_2_ capture [27]. Whilst the preliminary assessment of these materials was carried out solely using the equilibrium properties, their adsorption kinetics (*i*.*e*., controlling mechanism, pore tortuosity) are not well understood, particularly for shaped forms that can be used in a practical separation process [28].

The key objective of this study is to characterize the adsorption kinetics of CO_2_ in binderless pellets of three cationic forms of Y-type zeolites, namely, zeolite H-Y, Na-Y, and TMA exchanged Na-Y. To this end, we deployed an integrated experimental and computational framework that encompasses preparation of the adsorbent pellets, textural and equilibrium characterization (Sect. 2), ZLC experiments (Sect. 2.4), rigorous modeling of the mass transport within the adsorbent pellets, and non-linear parameter estimation (Sect. 3) to extract the relevant adsorption kinetics. We discuss our findings in terms of textural and equilibrium properties.

## Materials and methods

### Materials and gases

Crystalline zeolite H-Y was obtained from Zeolyst (CBV 400) and Na-Y was obtained from NIST (reference material, RM 8850). TMA exchanged Na-Y (referred to as NaTMA-Y) was produced by carrying out cation exchange as described in our previous work [27]. The adsorbent materials and gases used in this study are summarized in Table 1. The powder forms of the adsorbents were pelletized by placing approximately ${80}\,\textrm{mg}$ of powder, without any binder, into a ${5}\,\textrm{mm}$ pellet die, which was then placed in a Specac Manual Hydraulic Press (Specac Limited, United Kingdom). The powder was pressed with a load of ${0.4}\,\textrm{t}$ (equivalent to ${200}\,\textrm{Mpa}$ of gauge pressure) on the die which was maintained for ${30}\,\textrm{s}$ to form cylindrical pellets with a diameter of ${5}\,\textrm{mm}$ and an approximate height of ${5}\,\textrm{mm}$. Following this, the samples were broken to produce individual particles of approximately $10-20~\textrm{mg}$ with an approximate characteristic diameter of 1.3 to ${1.9}\,\textrm{mm}$ for ZLC experiments. For all the characterization experiments, the samples were activated *ex-situ* by heating to ${623}\,\textrm{K}$ under vacuum for ${16}\,\textrm{h}$ prior to the measurements. For the ZLC experiments, the samples were additionally activated *in-situ* within the experimental cell under constant carrier gas flow (helium or nitrogen) at ${393}\,\textrm{K}$ for ${2}\,\textrm{h}$ before the experiments.

**Table 1:** Details of the raw adsorbent materials, reagents, and gases employed in this study, as provided by the manufacturer/supplier

Name	CAS Number	Source	Purity [${\%}$]
Zeolite H-Y powder (CBV400)	1318-02-1	Zeolyst	N/A
Zeolite Na-Y powder (RM8850)	1318-02-1	NIST	N/A
Zeolite NaTMA-Y powder	N/A	Cation-exchanged from Na-Y	N/A
Carbon dioxide (CO_2_)	124-38-9	BOC	99.995
Helium (He)	7440-59-7	BOC	99.999
Nitrogen (N_2_)	7727-37-9	BOC	99.999

### Textural properties

The key textural properties measured for this study are the crystal density, mean macropore radius, macropore void fraction, as well as micropore, mesopore, and macropore volumes of the adsorbents. The crystal density $$\rho _\textrm{s}$$$$[\textrm{kg} \,\textrm{m}^{-3}]$$ of the crystalline forms of the three zeolites were obtained via high-pressure helium gravimetry at $${393.15}\,\textrm{K}$$ as reported in our previous study [27]. The micro- and meso-porosity of the adsorbents were determined by low-pressure N_2_ physisorption measurements at $${77}\,\textrm{K}$$ using an Autosorb iQ (Quantachrome Instruments, United States) after *ex-situ* activation following the procedure described in Section 2.1. A non-local density functional theory (NLDFT) model, assuming cylindrical/spherical pores on silica/zeolite model was applied for the computation of the pore size distribution (PSD) by using the proprietary data analysis software (ASiQwin version 5.2, Quantachrome Instruments, United States). The PSD was then used to obtain the total micropore ($$v_\textrm{mic}$$$$[\textrm{cm}^3\,\textrm{g}^{-1}]$$) and mesopore volumes ($$v_\textrm{meso}$$$$[\textrm{cm}^3\,\textrm{g}^{-1}]$$) for the adsorbents [29]. The total micropore volume was defined as the cumulative pore volume in pore diameters below $${2}\,\textrm{nm}$$, and the total mesopore volume as the cumulative pore volume in pore diameters between 2 and $${50}\,\textrm{nm}$$.

The macroporosity of the three pelletized materials was measured via mercury intrusion porosimetry (MIP) using an AutoPore IV Series Mercury Porosimeter (Micromeritics Instrument Corporation, United States) in the pressure range of $${3.7 \times 10^{-2}}\,\textrm{bar}$$ to $${2.23\times 10^{3}}\,\textrm{bar}$$. The samples were activated *ex-situ* following the procedure described in Section 2.1 before these measurements. The measurement provides the volume of mercury intruded as a function of pressure, which upon applying Washburn’s equation can be expressed as pore throat radius. It is assumed here that all intruded pores are circular at the surface and cylindrical in the interior. The data at low pressure is used to estimate the envelope volume of the adsorbent per unit mass of adsorbent $$m_\textrm{ads}$$ ($$v_\textrm{p}$$$$[\textrm{cm}^3\,\textrm{g}^{-1}]$$), and the full PSD is used to obtain the macropore volume of the sample ($$v_\textrm{mac}$$$$[\textrm{cm}^3\,\textrm{g}^{-1}]$$), which is defined as the total volume of pores with a pore diameter exceeding $${50}\,\textrm{nm}$$. Following this, pellet void macropore fraction (excluding micro- and meso-pores) ($$\varepsilon _\textrm{p}$$ [-]), and the volume of solid ($$V_\textrm{s}$$ [$$\textrm{m}^3$$]) can be calculated using the following equations.

1 $$\begin{aligned} \varepsilon _\textrm{p}&= \frac{v_\textrm{mac}}{v_\textrm{p}} \end{aligned}$$

2 $$\begin{aligned} V_\textrm{s}&= \frac{m_\textrm{ads}}{\rho _\textrm{s}} \end{aligned}$$

The empirical macropore PSD as a function of pore width *W* was fit to the kernel distribution using the fitdist function in MATLAB R2023a (The Mathworks Inc., United States) to yield a non-parametric estimation of the probability distribution function for the experimental macropore size distribution, $f(W)=\frac{V(W)}{v_\textrm{mac}}$. Here *V*(*W*) is the differential macropore size distribution per unit mass of adsorbent ($[\textrm{cm}^3\,\textrm{g}^{-1}]$). *f*(*W*) can then be integrated over the macropore range to obtain *intrinsic volume averaged* parameters, *i*.*e*., theoretical diffusivity [30]. The mean macropore radius $\bar{r}_\textrm{p}$ was obtained from the mean of *f*(*W*) and the bandwidth of *f*(*W*) (the fixed standard deviation of all the normal distributions that compose the kernel distribution) was used to describe the spread of the distribution around $\bar{r}_\textrm{p}$.

The MIP data can also be used to compute the tortuosity ($\tau _\textrm{MIP}$ [-]) of the pore network of the material, which is computed assuming cylindrical pores by using the following correlation [31]:

3 $$\begin{aligned} \tau _\textrm{MIP} = (2.23 - 1.13v_\textrm{tot}\rho _\textrm{bulk}) \left( 0.92\left[ \frac{4}{S_\textrm{MIP}} \sum _{i=\textrm{1}}^{i_\textrm{max}} \frac{\Delta V_{\textrm{int},i}}{W_{i}}\right] \right) ^{2} \end{aligned}$$

where $$v_\textrm{tot}$$$$[\textrm{cm}^3\,\textrm{g}^{-1}]$$ is the total volume of mercury intruded into the sample up to the pressure which corresponds to the macropore region, $$\rho _\textrm{bulk}$$$$[\textrm{g}\, \textrm{cm}^{-3 }]$$ is the bulk density of the solid, $$S_\textrm{MIP}$$$$[\textrm{m}^{2}\,\textrm{g}^{-1}]$$ is the cumulative pore area in the macropores obtained from MIP, and $$\Delta V_{\textrm{int},i}$$$$[\textrm{m}^{3}\,\textrm{g}^{-1}]$$ is the volume of mercury intruded in the pore diameter interval $$W_\textrm{i}$$ [$$\textrm{m}$$]. The summation is done in the macropore range only, starting from the first data point $$\ge$$$${50}\,\textrm{nm}$$ and ending at the maximum measured $$W_\textrm{max}$$. As described below, ZLC experiments will also be used to estimate the tortuosity $$\tau _\textrm{mac}$$ (the ratio between a theoretical diffusivity and the measured effective diffusivity) providing a useful means of comparison against MIP data.

### Equilibrium adsorption isotherms

The CO_2_ adsorption equilibrium was measured at $288 \,\textrm{K},298\,\textrm{K}$ and $308\,\textrm{K}$ in the pressure range ${1 \times 10^{-3}}\,\textrm{bar}$ to ${1.013}\textrm{bar}$ with an Autosorb iQ (Quantachrome Instruments, United States). The equilibrium data was modeled using the dual-site Langmuir (DSL) model given by:

4 $$\begin{aligned} q_j^{*}&= \frac{q_{\textrm{sb},j}b_jc_j}{1+b_jc_j} + \frac{q_{\textrm{sd},j}d_jc_j}{1+d_jc_j} \nonumber \\ b_j&= b_{0,j}\exp \left( \frac{-\Delta U_{\textrm{b},j}}{\textrm{R}T}\right) \nonumber \\ d_j&= d_{0,j}\exp \left( \frac{-\Delta U_{\textrm{d},j}}{\textrm{R}T}\right) \nonumber \\ c_j&= \frac{Py_j}{RT} \end{aligned}$$

where $$q_j^{*}$$$$[\textrm{mol}\,\textrm{kg}^{-1}]$$ is the absolute amount of gas *j* adsorbed on the adsorbent, $$c_j$$$$[\textrm{mol}\,\textrm{m}^{-3}]$$ is the bulk phase gas concentration computed using the ideal gas law at a given gas mole fraction $$y_j$$ [–], an absolute pressure *P*$$[\textrm{pa}]$$ and an absolute temperature *T*$$[\textrm{K}]$$, R $$[\textrm{J}\,\textrm{mol}^{-1}\,\textrm{K}^{-1}]$$ is the universal gas constant, $$q_{\textrm{sb},j}$$$$[\textrm{mol}\,\textrm{kg}^{-1}]$$ and $$q_{\textrm{sd},j}$$$$[\textrm{mol}\,\textrm{kg}^{-1}]$$ are the saturation capacities for the two adsorption sites, $$b_j$$$$[\textrm{m}^3\,\textrm{mol}^{-1}]$$ and $$d_j$$$$[\textrm{m}^3\,\textrm{mol}^{-1}]$$ are the temperature dependent adsorption coefficients, described by two constants each, $$b_{0,j}$$$$[\textrm{m}^3\,\textrm{mol}^{-1}]$$ and $$-\Delta U_{b,j}$$$$[\textrm{J}\,\textrm{mol}^{-1}]$$, and $$d_{0,j}$$$$[\textrm{m}^3\,\textrm{mol}^{-1}]$$ and $$-\Delta U_{d,j}$$$$[\textrm{J}\,\textrm{mol}^{-1}]$$ for the first and second sites respectively. Applying the Clausius-Clapeyron equation, the isosteric heat of adsorption $$|\Delta H_\textrm{is}|$$ is expressed as a function of the equilibrium adsorbed amount through the following expression [32]:

5 $$\begin{aligned}|\Delta H_\textrm{is}|= \left| \frac{\frac{q_{\textrm{sb},j} b_j \Delta U_{\textrm{b},j}}{\left( 1+b_j c_j\right) ^2}+\frac{q_{\textrm{sd},j} d_j \Delta U_{\textrm{d},j}}{\left( 1+d_j c_j\right) ^2}}{\frac{q_{\textrm{sb},j} b_j}{\left( 1+b_j c_j\right) ^2}+\frac{q_{\textrm{sd},j} d_j}{\left( 1+d_j c_j\right) ^2}}\right| \end{aligned}$$

The limiting heat of adsorption at zero-loading, $|\Delta H_\textrm{is,0}|$, is thus given by setting $c_j=0$. Using the DSL formulation, one can also extract the dimensionless Henry’s law constant, as given by

6 $$\begin{aligned} K_0 = \rho _\textrm{s}(q_{\textrm{sb},j}b_j+q_{\textrm{sd},j}d_j) \end{aligned}$$

The Henry’s constant computed using this expression is “dimensionless” in the sense that the amount adsorbed and the bulk concentration are expressed per unit volume (solid volume and gas volume, respectively).

### Zero-length column (ZLC) experiments

**Fig. 1:**
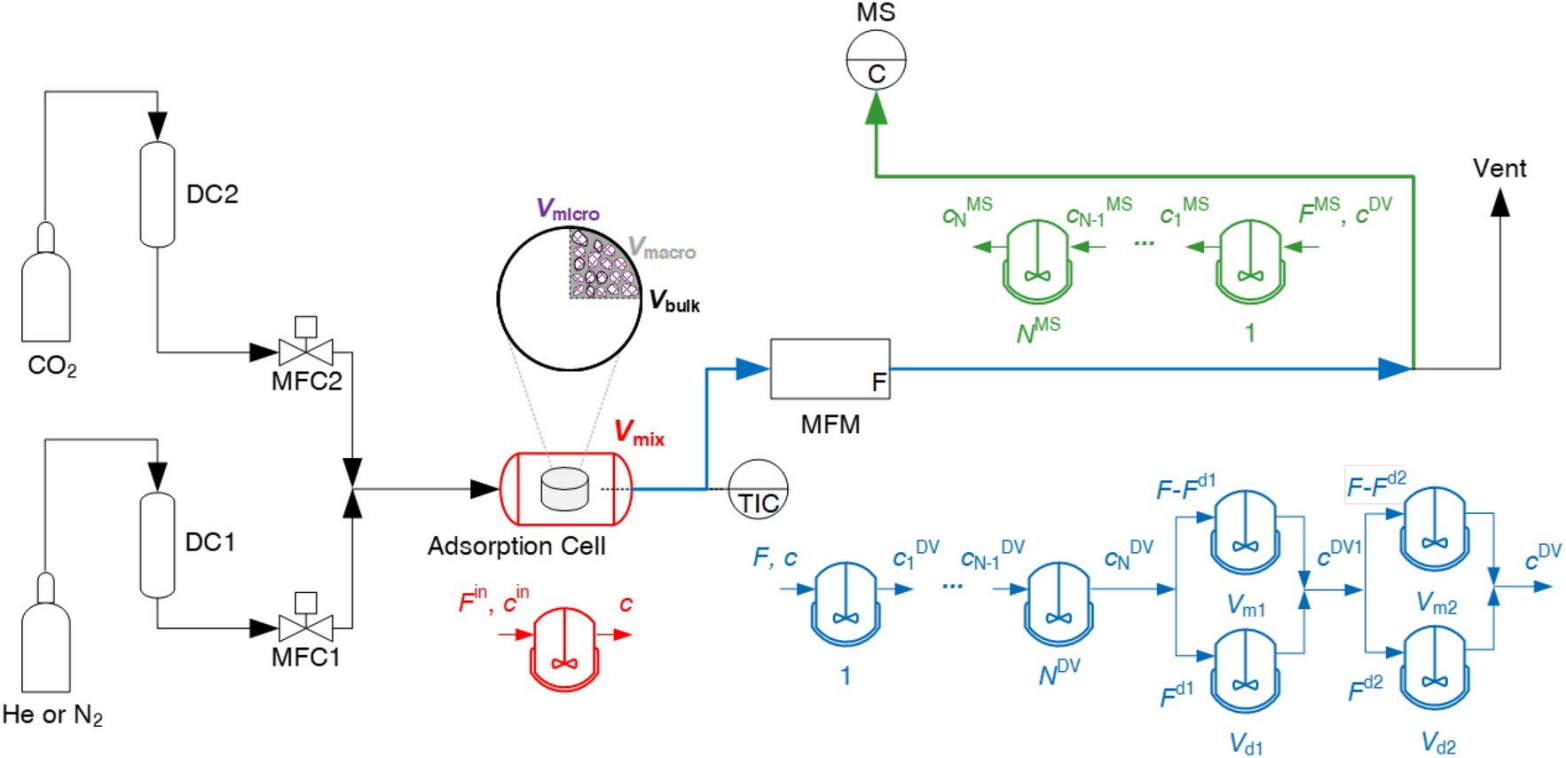
Schematic of the experimental setup used for the ZLC experiments

**Fig. 2:**
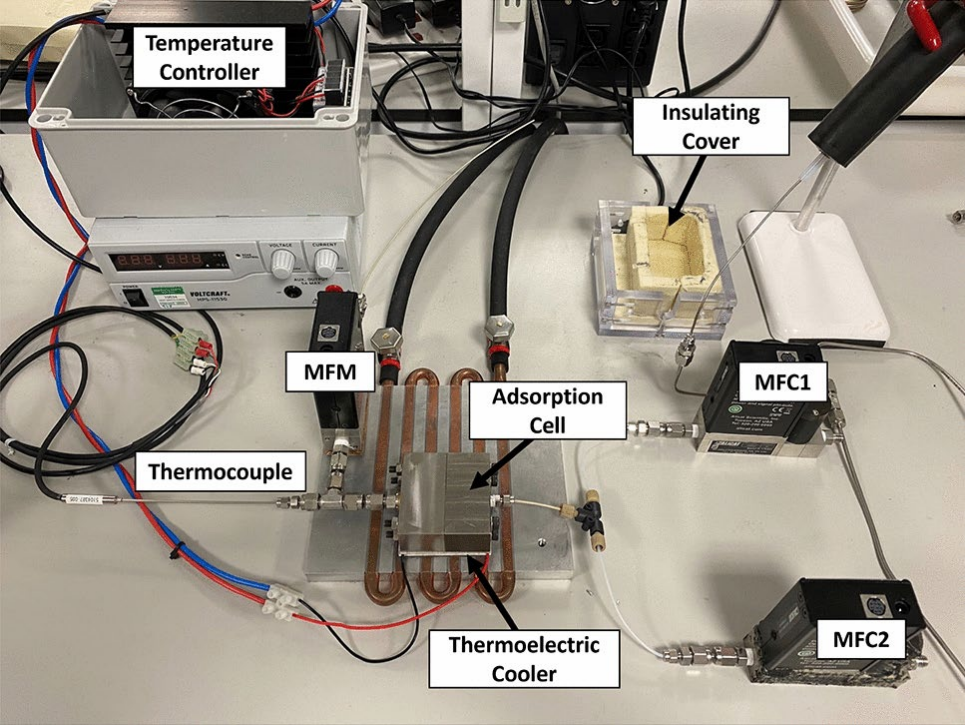
Top view of the experimental setup, described in Fig. 1, excluding the gas cylinders and drying columns (DC) of the gas supply system, and the mass spectrometer (MS).

The zero-length column (ZLC) experiments were carried out using an upgraded version of the apparatus developed in our previous work [25, 33]. Figure 1 shows a schematic of this setup and a photograph of the experimental setup is shown in Fig. 2.

The gas supply system (black) consists of helium (or nitrogen) and CO_2_ gas cylinders connected to mass flow controllers (MFC) 1 and 2 respectively, through drying columns (DC) which are packed with zeolite 4A beads and placed in a thermostat set at ${273}\textrm{K}$. The adsorption cell (red) is custom-built (see Fig. 2) and houses the adsorbent particle. The adsorption cell was built in-house and allows precise temperature control using a temperature indicating control system (TIC) in the range $5-120</span>^\circ{\textrm{C}}$ composed of a thermo-electric cooler (Laird ETX25-12-F1-6262-TA-W6, Laird Thermal Systems GmbH, Germany) along with an OEM Precision Temperature Controller (TEC-1090-HV, Meerstetter Engineering GmbH, Switzerland). The temperature indicating control (TIC) system was modified from our previous work [25] and now uses a single thermocouple to measure and control the internal temperature of the adsorption cell. The temperature sensor is an inline PT100 thermocouple (Miniature RTD Sensor - PT100, TC Ltd., United Kingdom) connected through a tee-connector at the outlet and is placed close to the adsorbent particle such that it records the gas temperature at the site of adsorption. The top and bottom flat surfaces of the adsorption cell ensure direct thermal contact with the thermoelectric cooler, to (1) increase the thermal mass of the cell to ensure good heat dissipation, and (2) allow the use of a flat thermoelectric Peltier element. During an experiment, the ambient-side temperature of the thermoelectric cooler is maintained at $20\,^\circ\textrm{C}$ using a 6-pass liquid cold plate heat-sink (Aavid, United States), which is supplied with water at $20\, ^\circ\textrm{C}$ from a circulating chiller (Huber Minichiller 300, Huber, Germany). The Peltier element is immobilized on the surface of the heat sink by placing it on a self-adhesive graphite-based thermal interface sheet (RS PRO, United Kingdom). The thermal contact between the adsorption cell and the Peltier element is further enhanced by applying silicone thermal grease (RS PRO, United Kingdom) on its top surface prior to an experiment. The adsorption cell is insulated using a foam cover that is placed on the exposed surface of the cell. Tests were carried out by switching flow on and off and changing the flowrate from 50 to ${70}\,\textrm{cm}^3 \,\textrm{min}^{-1}$ under constant temperature control to ensure that the gas flow is at the same temperature as the setpoint temperature. No noticeable deviations from the setpoint were measured in these tests. The effluent flowrate from the adsorption cell *F* is measured using an in-line mass flow meter (MFM).

The internal volume of the adsorption cell $V_\textrm{cell}$ is cylindrical with a volume of ${0.79}\,\textrm{cm}^{3}$. The empty volume of the setup including the blank volume within the adsorption cell and the volume of the lines connecting it to the mass spectrometer (MS) corresponds to the blank volume of the apparatus (blue and green respectively). The adsorption cell and the blank volume are modeled as connected continuous stirred tank reactors (CSTRs), as indicated underneath each section with their corresponding volumes, flow rates, and gas inlet/outlet concentrations.

Following sample activation as described in Sect. 2.1, the experiments were carried out as follows:

1. Under constant carrier gas flow, the adsorbent particle was degassed *in situ* at ${393.15}\textrm{K}$ for 2 h,
2. Under constant carrier gas flow, the temperature control system was set to the experimental temperature ($T_\textrm{0}$), in the range ${278.15}\,\textrm{K}$ to ${393.15}\,\textrm{K}$.
3. Following thermal equilibration, the adsorbent was saturated at a given CO_2_ composition ($y^\textrm{0}_\mathrm {CO_2}$) using a mixture of CO_2_ and the carrier gas at a constant flow rate at ambient pressure (*P*).
4. Once the adsorbent was fully saturated, the gas flow was switched to the carrier gas at a given flow rate ($F^\textrm{in}$), and the timer for the desorption experiment was commenced.
5. The composition of the gas at the MS ($y_{\textrm{CO}_{2}}$), the temperature of the adsorption cell (*T*), and the exit flow rate from the adsorption cell (*F*) were tracked until the completion of the experiment.

For each of the adsorbents, separate experiments were carried out using helium and nitrogen as the carrier gas at three different carrier gas flow rates, $F^\textrm{in}$ = 50, 60, and ${70}\,\textrm{cm}^3 \,\textrm{min}^{-1}$. The experiments using helium as a carrier gas were carried out at three temperatures, ${288.15\,\textrm{K},298.15\, \textrm{K}}$ and $308.15\,\textrm{K}$, while the experiments using nitrogen as a carrier gas were carried out at ${288.15}\,\textrm{K}$. In all the experiments, the adsorbents were saturated with CO_2_ at a partial pressure of $p = Py^\textrm{0}_\mathrm {CO_2} = {0.10}\,{bar}$.

The procedure above was repeated using both carrier gases at ambient temperature with an empty adsorption cell to obtain the blank cell response corresponding to the desorption experiments. The blank responses were fit to a model that describes the physical mixing, advection, and diffusion in the tubing downstream of the adsorbent particle followed by the flow through the capillary tube of the mass spectrometer. The fitted model is used to propagate the ZLC response at the outer boundary of the adsorbent particle obtained from solving the model described in Section 3.1 to predict the composition that would be measured by the detector (MS).

## Mathematical framework

### Zero-length column (ZLC) model

The adsorbent is described as a well-mixed CSTR based on the assumption of a zero-length column (ZLC) [19]. This assumption holds for a sufficiently small single adsorbent pellet as the axial length of the adsorbent tends to zero. Additionally, the desorption of a single adsorbing component *j* is assumed to be isothermal and isobaric yielding the component material balance

7 $$\begin{aligned} F^\textrm{in}c_{j}^\textrm{b,in} - Fc_{j}^\textrm{b} = V_\textrm{mix}\frac{\partial c_{j}^\textrm{b}}{\partial {t}} + V_\textrm{p}\frac{\partial {\bar{n}_j}}{\partial {t}} \end{aligned}$$

where $$F^\textrm{in}$$$$[\textrm{m}\,^{3}\,\textrm{s}^{-1}]$$ and *F*$$[\textrm{m}\,^{3}\,\textrm{s}^{-1}]$$ are the volumetric mixture gas flow rate at the inlet and the outlet of the adsorption cell, respectively, $$c_{j}^\textrm{b,in}$$$$[\textrm{mol}\,\textrm{m}^{-3}]$$ and $$c_{j}^\textrm{b}$$$$[\textrm{mol}\,\textrm{m}^{-3}]$$ are the bulk concentration of the gas *j* at inlet and outlet of the adsorption cell, respectively, $$y^\textrm{b}_j$$ is the mole fraction of *j* in the bulk [-], *T* is the absolute temperature $$[\textrm{K}]$$, $$V_\textrm{p}$$ [$$\textrm{m}^3$$] is the total volume of the adsorbent, and $$V_\textrm{mix}$$ [$$\textrm{m}^3$$] is taken to be the internal volume of the adsorption cell minus the volume of the adsorbent particle (*i*.*e*., $$V_\textrm{mix} = V_\textrm{cell}-V_\textrm{p}$$). $${\partial {\bar{n}_j}}/{\partial {t}}$$ is the rate of accumulation of the adsorbing component *j* within the adsorbent. This term is proportional to the molar flux of component *j* at the external surface of the adsorbent particle and can be expressed using a volume-averaged concentration ($$\bar{c}_{j}^\textrm{m}$$) and the solid loading ($$\bar{q}_{j}$$) within the spherical adsorbent particle of radius $$R_{\textrm{p}}$$:

8 $$\begin{aligned} \frac{\partial {\bar{n_j}}}{\partial {t}} = \varepsilon _{\textrm{p}} \frac{\partial \bar{c}_{j}^\textrm{m}}{\partial {t}} + (1-\varepsilon _{\textrm{p}}) \frac{\partial \bar{q}_{j}}{\partial {t}} \end{aligned}$$

where,

9 $$\begin{aligned} \frac{\partial \bar{c}_{j}^\textrm{m}}{\partial {t}}&= \frac{3}{R_{\textrm{p}}^3} \int _0^{R_{\textrm{p}}} \frac{\partial c_{j}^\textrm{m}}{\partial {t}} r^2 \mathrm {~d} r \end{aligned}$$

10 $$\begin{aligned} \frac{\partial \bar{q}_{j}}{\partial {t}}&= \frac{3}{R_{\textrm{p}}^3} \int _0^{R_{\textrm{p}}} \frac{\partial q_{j}}{\partial {t}} r^2 \mathrm {~d} r \end{aligned}$$

We note that in this formulation, $\varepsilon _\textrm{p}$ is the porosity of the adsorbent particle excluding micropores and mesopores; $c_{j}^\textrm{m}$ is the molar gas phase concentration of *j* in the macropores at the radial coordinate *r*, and ${q_j}$ is the solid phase loading of *j* at the radial coordinate *r*.

Assuming an ideal binary gas mixture at total pressure *P*, the bulk concentration of adsorbing component *j* can be expressed in terms of its mole fraction $y^\textrm{b}_j$:

11 $$\begin{aligned} c^\textrm{b}_{j} = \frac{Py^\textrm{b}_j}{RT} \end{aligned}$$

Following this, the mass conservation constraint is imposed to close the system of equations:

12 $$\begin{aligned} \sum \limits _{j=1}^{2}y^\textrm{b}_j = 1 \end{aligned}$$

This set of equations gives a general formulation for the ZLC model and the solution of ${\partial {\bar{n}_j}}/{\partial {t}}$ in Eq. 8 depends on the choice of mass transfer model used to describe the kinetics within the adsorbent, as discussed in Sect. 3.2.

### Mass balance around the adsorbent

In this work, we describe the mass balance around the adsorbent particle by using a model that assumes 1-D radial diffusion in the macropores of the adsorbent and the linear driving force (LDF) formulation to describe the rate of uptake of adsorptive into the adsorbent crystals. By assuming a spherical control volume and applying Fick’s first law, the following mass balance around the adsorbent particle is obtained:

13 $$\begin{aligned} \begin{aligned} \varepsilon _\textrm{p} \frac{\partial c_{j}^\textrm{m}(r,t)}{\partial {t}} + (1-\varepsilon _\textrm{p}) \frac{\partial {{q_j(r,t)}}}{\partial {t}} - \frac{1}{r^2} \frac{\partial }{\partial {r}}\left( D_\textrm{mac}^\textrm{e}r^2 \frac{\partial c_{j}^\textrm{m}(r,t)}{\partial {r}} \right) = 0,\\ 0< r < R_\textrm{p} \end{aligned} \end{aligned}$$

where $D_\textrm{mac}^\textrm{e}$ is the effective macropore diffusivity. For Eq. 13, the following boundary conditions apply at the center ($r=0$) and at the external surface of the adsorbent particle ($r=R_\textrm{p}$):

14 $$\begin{aligned} & \left. \frac{\partial c_{j}^\textrm{m}(r,t)}{\partial {r}} \right| _{r=0} = 0, \;\;\; t> 0 \end{aligned}$$

15 $$\begin{aligned} & c_{j}^\textrm{b}(t) = c_{j}^\textrm{m}(R_\textrm{p},t), \;\;\; t> 0 \end{aligned}$$

In imposing the second boundary condition we have assumed that there is negligible film resistance between the bulk gas and the surface of the adsorbent. For a ZLC experiment carried out at low pressure and temperature, $D_\textrm{mac}^\textrm{e}$ is the effective macropore diffusivity described by a pore volume averaged theoretical diffusivity $D_\textrm{p}$ that accounts for contributions from primarily molecular diffusion ($D_{12}^\textrm{m}$) and Knudsen diffusion ($D_j^\textrm{K}$):

16 $$\begin{aligned} D_\textrm{mac}^\textrm{e} = \frac{\varepsilon _{\textrm{p}}D_\textrm{p}}{\tau _\textrm{mac}} \end{aligned}$$

where $\tau _\textrm{mac}$ is defined here as the tortuosity of the macropore network of the adsorbent particle. As described in the following, the only unknown parameter in Eq. 16 is $\tau _\textrm{mac}$, which can thus be estimated from the experimental ZLC response curves and used to calculate the $D_\textrm{mac}^\textrm{e}$ for any gas mixture.

We describe $D_\textrm{p}$ using the parallel pore model proposed by Wang and Smith [34, 35] which, for the probability distribution function of a known macropore pore size distribution *f*(*W*) is given by

17 $$\begin{aligned} \varepsilon _{\textrm{p}}D_\textrm{p} = \int _{{50}\,\textrm{nm}}^{W_\textrm{max}} D_j(W)f(W) \,dW \end{aligned}$$

Here, the macropore size distribution is volume averaged over the pore width range greater than ${50}\,\textrm{nm}$. $D_j(W)$ is the theoretical diffusivity of the adsorbate as a function of *W* which is evaluated over the entire macropore size distribution. For low-pressure experiments with a single adsorbing species $D_j(W)$ is given by the Bosanquet equation [36] as the inverse of the reciprocal sum of Knudsen and molecular (bulk) diffusivity:

18 $$\begin{aligned} D_j(W) = \left( \frac{1}{D_{12}^{\textrm{m}}}+\frac{1}{D_j^{\textrm{K}}(W)}\right) ^{-1} \end{aligned}$$

For Knudsen diffusion, intermolecular interactions can be neglected and $D_j^{\textrm{K}}(W)$ for component *j* is calculated using kinetic theory upon applying the Derjaguin’s correction [37]:

19 $$\begin{aligned} D_j^{\textrm{K}}(W) = \frac{3}{13} W \sqrt{\frac{8 R T}{\pi M_j}} \end{aligned}$$

The molecular diffusivity $D_{12}^{\textrm{m}}$ is calculated assuming steady-state equimolar counter diffusion of a binary mixture of *j* in the carrier gas (helium or nitrogen) using the Chapman-Enskog equation [38]:

20 $$\begin{aligned} D_{12}^{\textrm{m}} = \frac{0.001858 T^{1.5}\sqrt{\left( \frac{1}{M_1}+\frac{1}{M_2}\right) }}{P \sigma _{12}^2 \Omega _{D, 12}} \end{aligned}$$

where subscripts 1 and 2 correspond to the component *j* and the carrier gas respectively, *M* is the molecular weight [$\textrm{kg}\, \textrm{mol}^{-1}$], *P* is the total pressure [atm], $\sigma$ is the collision diameter obtained from the Lennard-Jones potential [Å], $\Omega$ is the collision integral for the pair of molecules and was obtained from data tabulated in Bird et al. [38]. All parameter values are given in Table S1 in the Supporting Information.

**Fig. 3:**
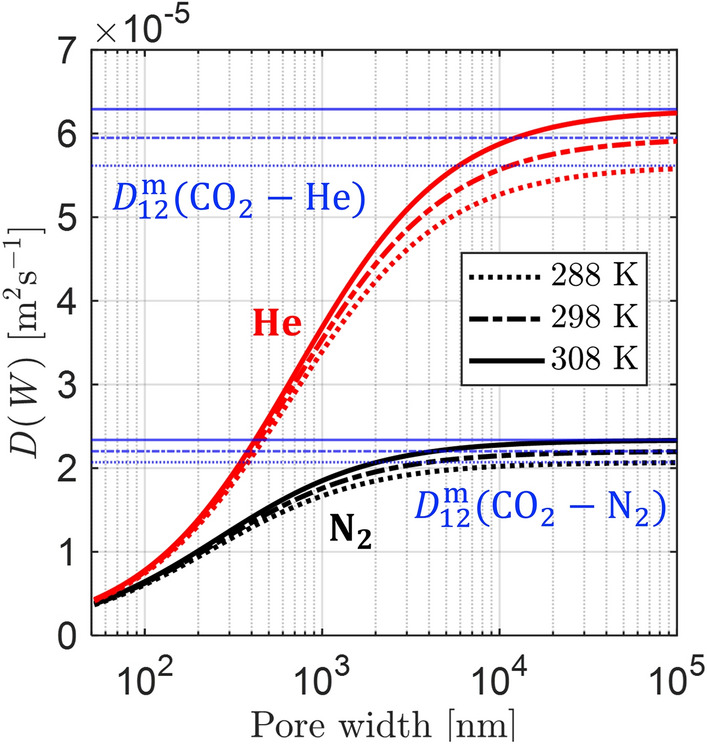
Theoretical diffusivity of the CO_2_ in helium **(red)** and nitrogen **(black)** in the macropore region as a function of pore width *W*, given by Eq. 18, evaluated at the three temperatures as the ZLC experiments. The blue horizontal lines correspond to the steady-state equimolar counter diffusivity $D_{12}^{\textrm{m}}$ for CO_2_ in helium and nitrogen at the respective temperatures.

In addition its dependence on the pore width *W*, $D_j(W)$ also depends on the gas mixture and the temperature, as shown in Fig. 3 for CO_2_ in helium (red curves) and nitrogen (black curves). At small pore sizes, the overall diffusion is dominated by Knudsen diffusion which is independent of the carrier gas properties, while at sufficiently large pore widths, $D_j(W)$ approaches the diffusivity of the bulk fluid (*i*.*e*., $D_{12}^{\textrm{m}}$). The temperature dependence becomes stronger as molecular diffusion dominates the overall process. The parallel pore model accounts for the distribution in pore size as it is physically equivalent to modeling the diffusion process as occurring in a collection of parallel pores distributed in frequency by the respective PSD. Thus when applying the parallel pore model (by combining $D_j(W)$ and *f*(*W*)), the upper limit for the effective macropore diffusivity ($D_\textrm{mac}^\textrm{e} = \frac{\varepsilon _{\textrm{p}}D_\textrm{p}}{\tau }$) is also bound to the bulk diffusivity of the gas.

This model is preferred for modeling diffusion in pore networks that exhibit wide pore size distributions. The commonly used approach of using a single equivalent pore size for the entire macropore network, *i*.*e*., applying the Bosanquet equation (Eq. 18) directly using the mean pore width as proposed by Satterfield [39], can result in unrealistically low values for the tortuosity for such materials [34]. This will be discussed further in Sect. 5.3

The material balance at the surface of the adsorbent crystals is described in terms of the volume-averaged loading within a crystal ($q_j$) at the radial coordinate *r* by the linear driving force (LDF) model:

21 $$\begin{aligned} \begin{aligned} \frac{\partial {{q_j(r,t)}}}{\partial {t}} = \frac{15D_\mathrm {\mu }^\textrm{e}}{r_\textrm{c}^2}\left( q_j^* - {q_j(r,t)}\right), \\ 0< r < R_\textrm{p} \end{aligned} \end{aligned}$$

where $q_j^*$ is the equilibrium adsorption capacity at $c_{j}^\textrm{m}$, obtained from Equation 4, and $D_\mathrm {\mu }^\textrm{e}/r_\textrm{c}^2$ is a reciprocal diffusion time constant expressed in terms of the radius of the adsorbent crystals, $r_\textrm{c}$, and the effective micropore diffusivity, $D_\mathrm {\mu }^\textrm{e}$. Assuming no film resistance at the surface of the crystal and upon application of the Glueckauf approximation [40], Equation 21 can also be expressed using the corresponding LDF mass transfer coefficient for the given gas *j*, $k^\mathrm {\mu }_j=5D_\mathrm {\mu }^\textrm{e}/r_\textrm{c}$.

As aforementioned, the initial conditions for a desorption experiment are that of complete adsorption and thermal equilibrium between the bulk gas and the adsorbent, before the gas flow is switched to the carrier gas at $t=0$.

22 $$\begin{aligned} & c_{j}^\textrm{m}(r,0) = c^\textrm{b}_{j,0}, \;\;\; 0< r < R_\textrm{p} \end{aligned}$$

23 $$\begin{aligned} & q_{j}(r,0) = q^*_{j}(c^\textrm{b}_{j,0},T), \;\;\; 0< r < R_\textrm{p} \end{aligned}$$

Here the subscript “0” denotes the conditions at the start of the desorption experiment. Given the experiment is operated isothermally, the temperature remains constant at the set value throughout.

The combined mass balances, Eqs. 13and 21, subject to the initial conditions, Eqs. 14 and 15, and boundary conditions, Eqs. 22 and 23, form a series of differential algebraic equations (DAEs) that are discretized spatially along the radius of the particle ($R_\textrm{p}$) into a finite grid of 100 equally spaced grid points using a second-order finite difference discretization scheme. As the macropore diffusivity is independent of concentration and the model is isothermal, the method of lines was applied with the central difference discretization scheme. This reduces the DAEs into a system of ordinary differential equations (ODEs) that are solved numerically using a stiff solver in the integrate.ode function of the scipy package in Python 3.8.5.

### ZLC experiment design and verification

When carrying out ZLC experiments, certain checks need to be carried out to ensure the assumptions of kinetic control and isothermality are satisfied in the interpretation of the desorption curves [19]. These checks require the computation of two dimensionless numbers. The derivation of these numbers along with the conditions within which they satisfy the assumptions are discussed in detail elsewhere [19, 26].

The parameter *L* is used to validate the assumption of kinetic control, as it represents the ratio of the diffusion time constant and the time constant of the washout of the adsorbed phase. Mathematically,

24 $$\begin{aligned} L = \frac{1}{3} \frac{F^\textrm{in}}{K_\textrm{0}V_\textrm{s}}\frac{R_\textrm{p}^2}{D^{\textrm{e}}} \end{aligned}$$

where $K_\textrm{0}$ is the dimensionless Henry’s constant and $D^{\textrm{e}}$ is the effective diffusivity of the system. The derivation for $D^{\textrm{e}}$ in a macropore diffusion limited system is given in Section S5 in the Supporting Information. In this regime and upon substitution of Eq. 16 into Eq. 24, we obtain:

25 $$\begin{aligned} L_\textrm{mac} = \frac{1}{3} \frac{F^\textrm{in}}{V_\textrm{s}}\frac{R_\textrm{p}^2{\tau }}{{\varepsilon _{\textrm{p}}D_{\textrm{p}}}} \end{aligned}$$

For kinetic control $L_\textrm{mac}> 2$, whereby $L_\textrm{mac}> 5$ is ideal, as reported in previous literature [14].

The assumption of isothermality can be validated by computing the dimensionless number $\Gamma$, which accounts for contributions from three terms: $\alpha = \frac{|\Delta H_\textrm{0}|K_\textrm{0}c_\textrm{0}}{C_\textrm{s}T_\textrm{0}}$ which is the dimensionless adiabatic temperature change, $\delta =\frac{|\Delta H_\textrm{0}|}{R T_\textrm{0}}$ which accounts for the change in enthalpy, and $\gamma = \frac{ha/C_\textrm{s}}{F/K_\textrm{0}V_\textrm{s}}$ which accounts for the heat transfer between the solid and the bulk gas:

26 $$\begin{aligned} \Gamma = \frac{\alpha \delta }{\gamma } = \frac{|\Delta H_\textrm{0}|^2}{R T_\textrm{0}^2}\frac{F^\textrm{in}c_\textrm{0}}{3 \lambda V_\textrm{s}}R_\textrm{p}^2 \end{aligned}$$

Here, $$c_\textrm{0}$$ is the initial concentration of the sorbate at the start of desorption [$$\textrm{mol}\,\textrm{m}^{-3}$$], $$|\Delta H_\textrm{is, 0}|$$ is the heat of adsorption at $$c_\textrm{0}$$$$[\textrm{J}\,\textrm{mol}^{-1}]$$, $$T_\textrm{0}$$ [$$\textrm{K}$$] is the temperature at the start of desorption, *h* is the heat transfer coefficient between the adsorbent and the bulk gas $$[\textrm{J}\,\textrm{m}^{-2}\,\textrm{K}^{-1}\,\textrm{s}^{-1}]$$ (assumed to be $$h=\lambda /R_\textrm{p}$$, where $$\lambda$$ is the thermal conductivity of the carrier gas $$[\textrm{J}\,\textrm{m}^{-1}\,\textrm{K}^{-1}\,\textrm{s}^{-1}]$$), *a* is the external surface area to volume ratio of the adsorbent particle [$$\textrm{m}^{-1}$$] ($$a=3/R_\textrm{p}$$ for a spherical geometry), and $$C_\textrm{s}$$ is the volumetric heat capacity of the adsorbent $$[\textrm{J}\,\textrm{m}^{-3}\,\textrm{K}^{-1}]$$. To ensure isothermality, $$\Gamma <\Gamma _\textrm{max}$$, whereby $$\Gamma _\textrm{max}$$ depends on the value of $$L_\textrm{mac}$$, as reducing the mass transfer resistance between the adsorbent and the bulk gas reduces heat effects in the system. In a ZLC experiment, $$\Gamma _\textrm{max}$$ increases with $$L_\textrm{mac}$$ in proportion to $$R_\textrm{p}^2$$ [26].

**Table 2:** Parameters used to calculate dimensionless numbers *L* and $\Gamma$ in order to test experimental assumptions.

Material	$m_\textrm{ads}$	$V_\textrm{s}$	$R_\textrm{p}$	$|\Delta H_\textrm{is, 0}|$	$c_\textrm{0}$	$F^\textrm{in}$	$\lambda$
	[$\textrm{mg}$]	[$\textrm{cm}^{3}$]	[$\textrm{mm}$]	[$\textrm{kJ}\,\textrm{mol}^{-1}$]	[$\textrm{mol}\,\textrm{m}^{-3}$]	[$\textrm{cm}^{3}\,\textrm{s}^{-1}$]	[$\textrm{W}\,\textrm{m}^{-1}\,\textrm{K}^{-1}$]
H-Y	26	0.012	1.89	26.2	4.40	0.83 / 1.00 / 1.17	0.15
Na-Y	13	0.005	1.34	30.9			
NaTMA-Y	20	0.009	1.54	29.0

**Table 3:** Dimensionless parameters $$L_\textrm{mac}$$ and $$\Gamma$$ calculated for the experiments carried out at $$T =$$$${288.15}\,\textrm{K}$$, and $$c_0 =$$$${4.40}\,\textrm{mol}\,\textrm{m}^{-3}$$. $$\Gamma _\textrm{max}$$ was obtained for each value of $$L_\textrm{mac}$$ from the literature [26].

Material	$L_\textrm{mac}$	$\Gamma _\textrm{max}$	$\Gamma$	$L_\textrm{mac}$	$\Gamma _\textrm{max}$	$\Gamma$	$L_\textrm{mac}$	$\Gamma _\textrm{max}$	$\Gamma$
	50$\textrm{cm}^{3}\,\textrm{min}^{-1}$			60$\textrm{cm}^{3}\,\textrm{min}^{-1}$			70$\textrm{cm}^{3}\,\textrm{min}^{-1}$		
H-Y	11.9	6.7	2.3	14.3	8.4	2.8	16.7	10.4	3.3
Na-Y	11.0	6.1	3.8	13.2	7.5	4.6	15.4	9.1	5.4
NaTMA-Y	8.2	4.1	2.5	10.2	5.3	3.0	11.9	6.7	3.5

In designing our experiments, the dimensionless numbers $L_\textrm{mac}$ and $\Gamma$ were calculated for the three adsorbents studied. The relevant experimental parameters used for this computation are summarised in Table 2, and the resulting values are summarised in Table 3. In this exercise, we have used a heuristic value for the tortuosity ($\tau = 2$) and the obtained $L_\textrm{mac}$ values have been used to estimate $\Gamma _\textrm{max}$ from reported values in the literature [26]. We can see that the conditions for the assumption of isothermality ($\Gamma < \Gamma _\textrm{max}$) and kinetic control ($L_\textrm{mac}> 5$) are satisfied at all experimental conditions.

**Fig. 4:**
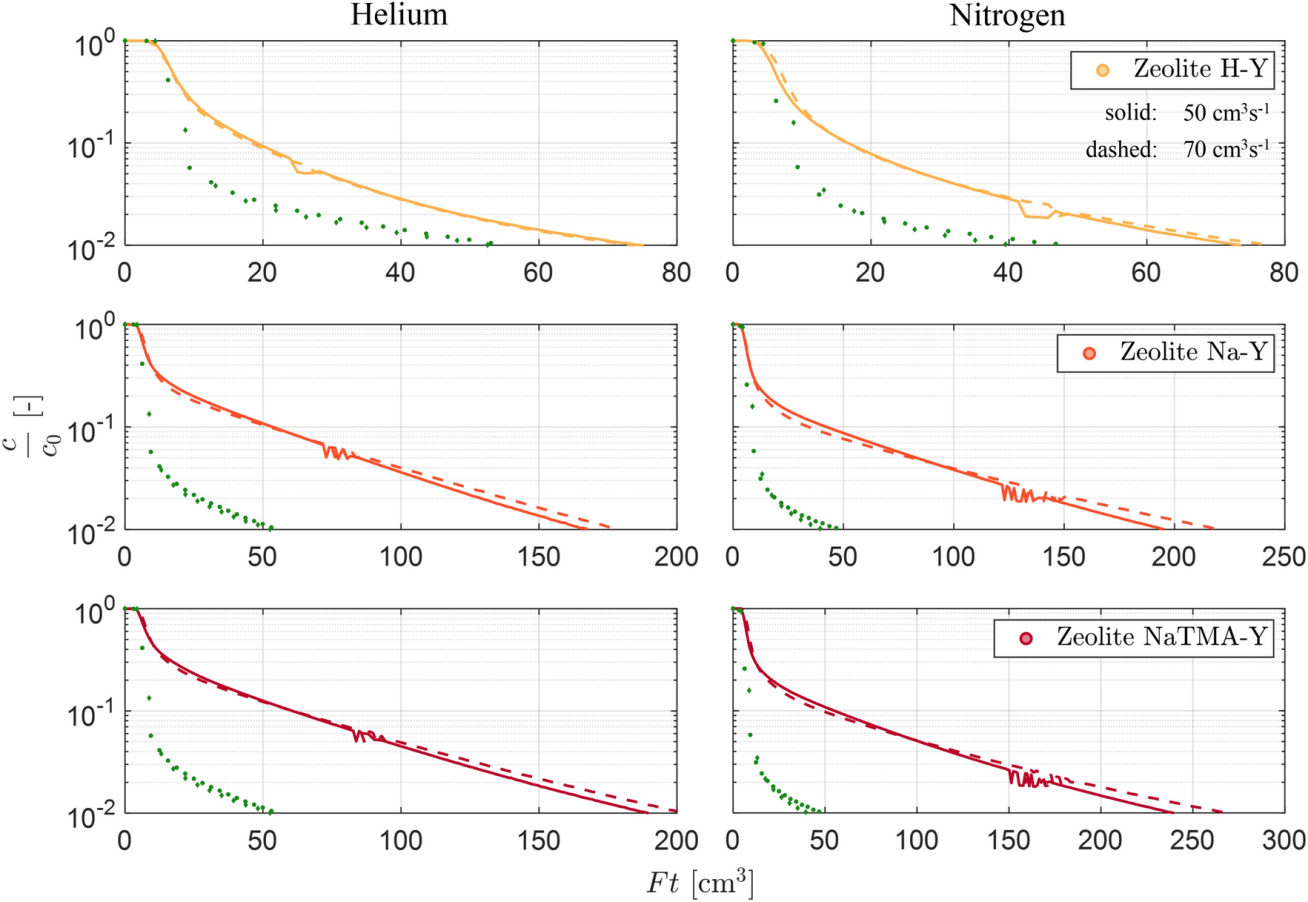
Experimental *Ft* plots using two different carrier gases, helium (left) and nitrogen (right), for Zeolite H-Y, Na-Y, and NaTMA-Y at ${288.15}\,\textrm{K}$ at $F^\textrm{in} = {50}\,\textrm{cm}^{3}\,\textrm{min}^{-1}$**(**solid lines**)**, and $F^\textrm{in} = {70} \,\textrm{cm}^{3}\,\textrm{min}^{-1}$ (dashed lines) at $y_0 = 0.10$. Straight lines connect the discrete experimental data points for clarity. The green symbols represent the experimental blank responses of the setup at the respective flow rates.

As a first step upon obtaining the time-resolved ZLC response curves, graphical checks were done to confirm that the experiments were conducted under kinetic control. Figure 4 shows experimental *Ft* plots for Zeolite H-Y, Na-Y, and NaTMA-Y at ${288.15}\,\textrm{K}$ at the lowest and highest flowrates evaluated using helium and nitrogen as carrier gases. For the experiments to be under kinetic control, the *Ft* curves at different flow rates should cross and diverge. This is clearly seen in the ZLC responses for Zeolites Na-Y and NaTMA-Y using both carrier gases. However, the *Ft* curves do not appear to cross for Zeolite H-Y within the range of composition measured. This is likely a consequence of the low equilibrium capacity for H-Y at the initial partial pressure used to saturate the adsorbent and the fast kinetics of CO_2_ sorption in zeolites.

**Fig. 5:**
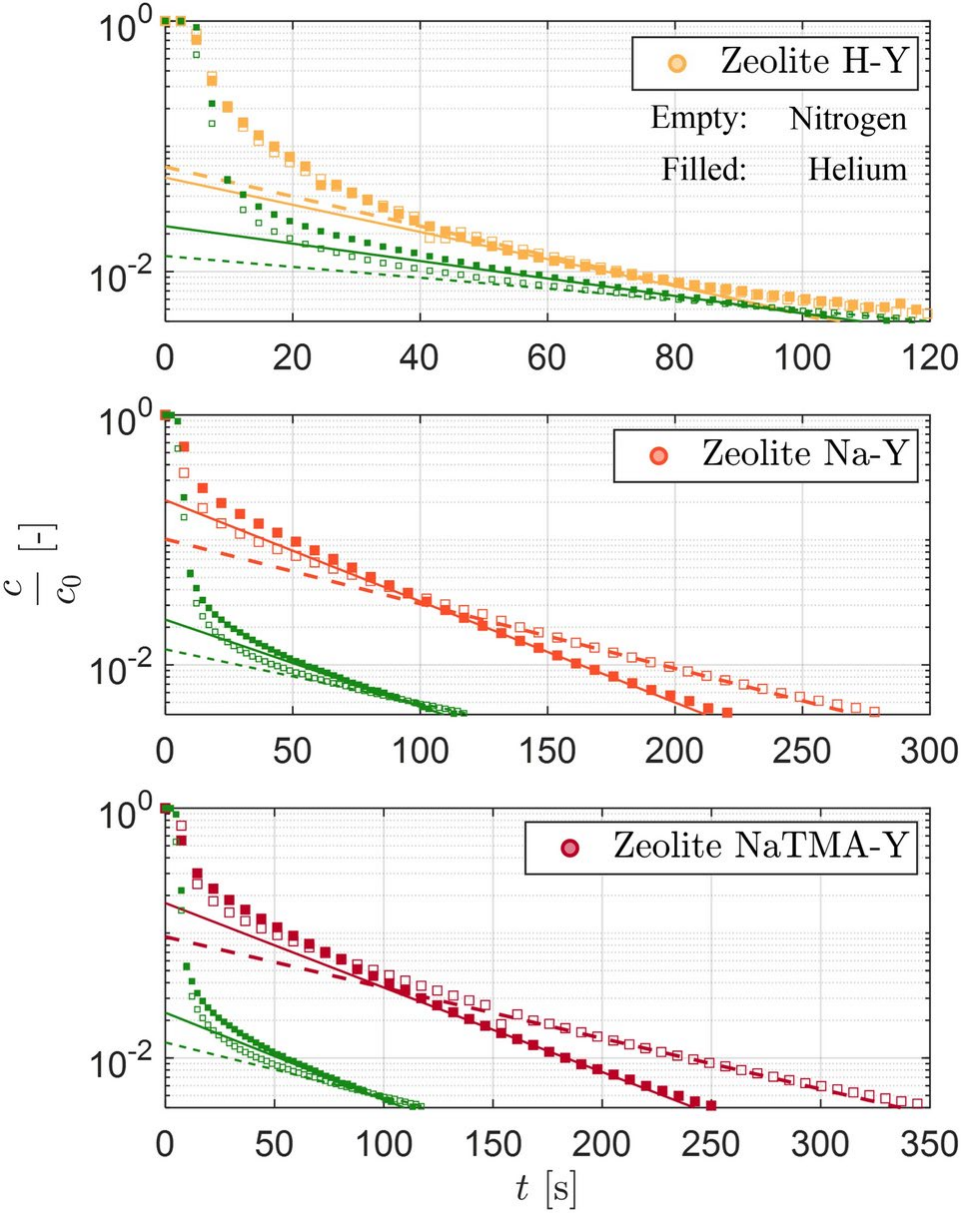
Experimental ZLC responses obtained using two different carrier gases, helium filled and nitrogen (empty), for Zeolite H-Y, Na-Y, and NaTMA-Y at ${288.15}\,\textrm{K}$ at $F^\textrm{in} = {60}\,\textrm{cm}^{3}\,\textrm{min}^{-1}$. The solid and dashed colored lines are linear fits to the long-time region of the ZLC curves for helium and nitrogen respectively. The green symbols correspond to the blank responses for each carrier gas at the same flow rate. The green solid and dashed lines are linear fits to the long-time region of the blank responses for helium and nitrogen respectively. For the ZLC responses, every 10th data point is shown for H-Y, and every 20th data point is for Na-Y and NaTMA-Y for clarity.

Next, graphical checks were carried out to determine the controlling mechanism for CO_2_ mass transport in these adsorbents. To this end, the time-resolved ZLC responses (composition against time) obtained using different carrier gases were compared by evaluating the slopes of the long-time asymptote on a logarithmic scale. For ZLC responses obtained using different carrier gases, a change in the slope is an indicator of macropore diffusion-controlled mass transport within the adsorbent particle. Figure 5 shows the comparison of the time-resolved ZLC responses using helium and nitrogen as the carrier gas at ${288.15}\,\textrm{K}$ and at a flowrate of ${60}\, \textrm{cm}^{3}\,\textrm{min}^{-1}$. For both Na-Y and NaTMA-Y, we can clearly see that changing the carrier gas from helium to nitrogen has reduced the slope of the long-time asymptote. For H-Y, the slopes of the long-time asymptotes are similar and the curves overlap. This behavior is again a consequence of the system being too fast to exhibit the long-time asymptote. For systems such as H-Y, the extraction of kinetic information requires decoupling the desorption response from the blank column response, as the latter does also depend on the carrier gas used. The modeling of the blank response is described in Section 4.2.

### Parameter estimation

The goal of the parameter estimation is to fit the mass transfer model described in the previous section to the experimental ZLC response curves obtained from the procedure described in Sect. 2.4. The model (Sect. 3.1) describes two mechanisms for mass transport within an adsorbent: micropore and macropore diffusion. The recommendation for ZLC experiments that aim to fit such a model to experimental data is to first identify the controlling mass transport mechanism by carrying out additional experiments and to fit only the parameters required to describe the controlling mechanism [19]. The results in the previous section (Sect. 3.3) provided sufficient prior information to suggest that these systems are macropore diffusion-controlled.

For macropore diffusion-controlled systems, the diffusion within the crystals is much faster than that in the macropores. For such a system, it can be assumed that the adsorbed phase in the crystals is in local equilibrium with the bulk gas in the macropores. Thus, the intra-crystalline (micropore) diffusion parameter (${D_\mathrm {\mu }^\textrm{e}/r_\textrm{c}^2}$) should not have any impact on the shape of the modeled ZLC response. Numerically, this was enforced by fixing the micropore diffusion parameter to a high enough value ($\frac{D_\mathrm {\mu }^\textrm{e}}{r_\textrm{c}^2} = {20}\,\textrm{s}^{-1}$) beyond which the ZLC response is unaffected by its numerical value. This leaves the macropore tortuosity ($\tau _\textrm{mac,PP}$) as the only kinetic parameter that needs to be fitted (here, PP is used to indicate the application of the parallel pore model formulation, as opposed to using a single characteristic pore width, $\tau _\textrm{mac,mean}$, as further discussed in Section 5.3). The lower bound for the tortuosity was set to 0.1 although the minimum value that would be physically consistent for a porous adsorbent would be $\tau _\textrm{mac,PP} = 1$. A lower bound below this limit was chosen for the parameter estimation to determine that the model that describes the mass transport in the macropores using the parallel pore model formulation is appropriate for the systems studied. For instance, if the fitted value for $\tau _\textrm{mac,PP}$ is below 1, then the diffusion model chosen may not be suitable for the system. The upper bound was set to 10.

As for the fixed inputs, the adsorption equilibrium isotherms for CO_2_ were used as known inputs in the parameter estimation. The model parameters for the isotherms were obtained by fitting the dual-site Langmuir (DSL) model to the volumetric isotherms obtained using the procedure described in Sect. 2.2. In addition to this, the sample mass $m_\textrm{ads}$, crystal density $\rho _\textrm{s}$, and macropore void fraction $\varepsilon _\textrm{p}$ were used as fixed inputs. The single decision variable $\tau _\textrm{mac,PP}$ (which is assumed to be constant for each material) is used to compute an effective macropore diffusivity within the model according to Eq. 16 to carry out the optimization. The parallel pore model was used to determine the theoretical diffusivity ($\varepsilon _\textrm{p}D_\textrm{p}$), for CO_2_ in each of the two carrier gases, given each material’s macropore size distribution using Eq. 17. The parameter estimation for each adsorbent required fitting 9 experiments simultaneously (3 flow rates and 3 temperatures with helium as the carrier gas). The maximum likelihood estimator (MLE) approach was applied for the parameter estimation using an evolutionary optimization algorithm (geneticalgorithm2 [41] (v6.2.4) in Python 3.8.5) [25].

## Results

### Adsorbent characterization

#### Textural characterization

**Fig. 6:**
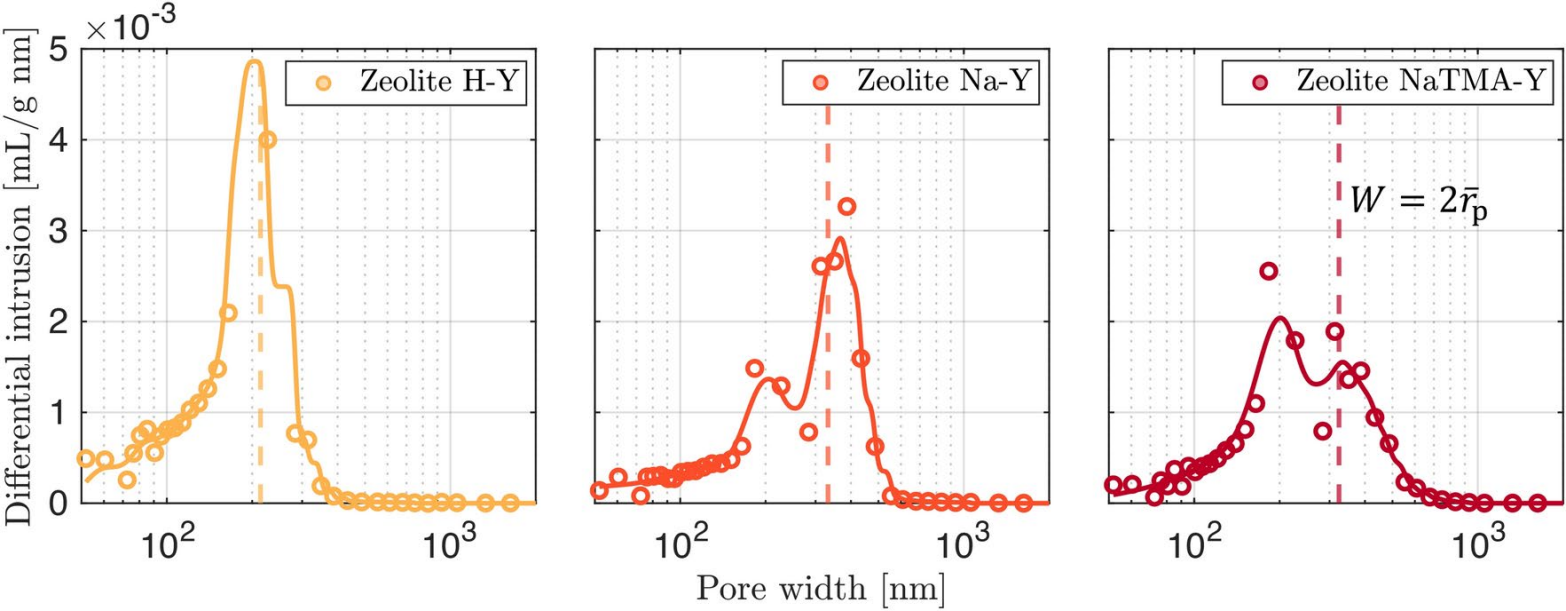
Pore size distribution in the macropore region for H-Y (yellow), Na-Y (orange), and NaTMA-Y (maroon) obtained by mercury intrusion porosimetry (MIP). The horizontal axis has been limited to the regions which shows intrusion. The solid colored lines show the fitted kernel distribution and the vertical dashed line corresponds to the mean macropore diameter ($2 \bar{r}_\textrm{p}$) for each material.

Figure 6 shows the pore-size distributions (PSD) in terms of differential intrusion of mercury into the adsorbent samples as a function of pore width in the macropore range (> $50\textrm{ nm}$). The colored solid lines correspond to the kernel distribution fitted to the experimental data. Overall, the kernel distribution provides a good qualitative fit throughout. The PSD for H-Y appears unimodal whilst both Na-Y and NaTMA-Y exhibit broad bimodal distributions in the macropore region. All three materials exhibit a peak in the distributions around ${200}\textrm{ nm}$ which could result from the identical pelletization routine being used for all three materials.

We also estimate the equivalent pore diameters ($$2 \bar{r}_\textrm{p}$$) from the mean of the kernel distribution (shown in Fig. 6 as vertical dashed lines). These values and the broadness of the fitted distributions (bandwidth of the kernel distribution) are reported in Table 4. The mean pore sizes for Na-Y and NaTMA-Y are very similar ($$\bar{r}_\textrm{p}=164$$$$\textrm{nm}$$), which is expected, given that NaTMA-Y is produced from cation exchange on Na-Y. However, the spread of the PSD for NaTMA-Y is wider than for Na-Y potentially due to the disruption of the crystal shape and structure during the cation exchange process. The mean macropore radius is significantly smaller for H-Y ($$\bar{r}_\textrm{p}=107$$$$\textrm{nm}$$), likely due to differences in the crystal size in comparison with Na-Y. The pore surface areas computed using the MIP data ($$S_\textrm{MIP}$$) show the expected inverse trend to the mean pore radius, *i*.*e*., a larger proportion of smaller pores would correspond to a higher surface area as seen for H-Y.

Lastly, we also note that the tortuosity values calculated using MIP data ($\tau _\textrm{MIP}$ = 1.62– 1.71) are smaller than what is reported in the literature for commercial FAU zeolites which are typically in the range of 2– 3 [23, 42]. A complete summary of the textural characterization of the materials is given in Table 4. The raw MIP data is given in Figure S2 in the Supporting Information. Section S3 in the Supporting Information discusses the micro- and meso-pore size distributions of the three materials.

**Table 4:** Textural properties of the adsorbent materials derived from helium gravimetry, N_2_ adsorption isotherms at ${77}\,\textrm{K}$, (reported in our previous work [27]) and mercury intrusion porosimetry. The value in parentheses for $\bar{r}_\textrm{p}$ represents the bandwidth of the kernel distributions fitted to the experimental PSDs.

Material	$\rho _\textrm{s}$	$v_\textrm{bulk}$	$v_\textrm{mic}$	$v_\textrm{meso}$	$v_\textrm{mac}$	$\varepsilon _\textrm{p}$	$S_\textrm{MIP}$	$S_\textrm{BET}$	$\bar{r}_\textrm{p}$	$\tau _\textrm{MIP}$
	$[\textrm{g}^3\,\textrm{cm}^{-3}]$	$[\textrm{cm}^3\,\textrm{g}^{-1}]$	$[\textrm{cm}^3\,\textrm{g}^{-1}]$	$[\textrm{cm}^3\,\textrm{g}^{-1}]$	$[\textrm{cm}^3\,\textrm{g}^{-1}]$	[${\%}$]	$[\textrm{m}^{2}\,\textrm{g}^{-1}]$	$[\textrm{m}^{2}\,\textrm{g}^{-1}]$	[$\textrm{nm}$]	[-]
H-Y	2.130	0.944	0.260	0.080	0.510	54	25.68	741	107 (8)	1.62
Na-Y	2.410	1.295	0.359	0.027	0.608	47	7.96	914	166 (16)	1.65
NaTMA-Y	2.310	1.224	0.344	0.021	0.524	43	7.50	883	162 (23)	1.71

#### CO_2_ adsorption equilibrium

**Fig. 7:**
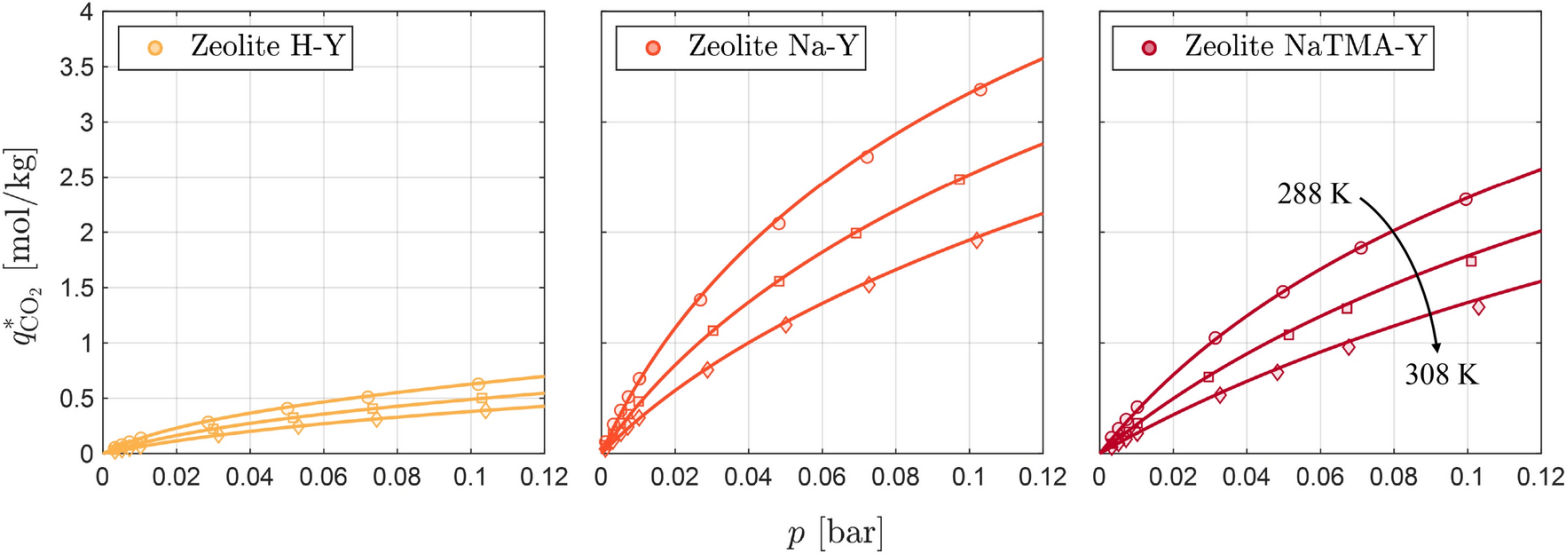
CO_2_ adsorption equilibrium isotherms for H-Y (yellow), Na-Y (orange), and NaTMA-Y (maroon) obtained by low-pressure volumetric measurements at $288.15\,\textrm{K},298.15\,\textrm{K}$ and $308.15\textrm{ K}$ (circles, squares, and diamonds respectively) in the pressure range evaluated in the ZLC experiments. The solid line through the measured points corresponds to the dual-site Langmuir (DSL) fit of the data given by Eq. 4. The complete isotherms measured up to $1\text{ bar}$ are presented in Figure S3 in the Supporting Information.

**Table 5:** Dual-Site Langmuir (DSL) isotherm model parameters derived from fitting the volumetric CO_2_ isotherms to Eq. 4. The values in parentheses represent the 95% confidence intervals obtained from the isotherm fitting.

Material	$q_\mathrm {sb,CO_{2}}$	$b_\mathrm {0,CO_{2}}$	$-\Delta U_\mathrm {b,CO_{2}}$	$q_\mathrm {sd,CO_{2}}$	$d_\mathrm {0,CO_{2}}$	$-\Delta U_\mathrm {d,CO_{2}}$
	$[\textrm{mol}\,\textrm{kg}^{-1}]$	[$\times 10^{-7}$ $\textrm{m}^{3}\,\textrm{mol}^{-1}$]	[$\textrm{kJ}\,\textrm{mol}^{-1}$]	$[\textrm{mol}\,\textrm{kg}^{-1}]$	[$\times 10^{-7}$ $\textrm{m}^{3}\,\textrm{mol}^{-1}$]	$[\textrm{kJ}\,\textrm{mol}^{-1}]$
H-Y	6.64 (0.03)	2.08 (0.01)	26.11 (0.01)	0.43 (0.00)	10.56 (0.63)	32.32 (0.15)
Na-Y	6.50 (0.02)	3.54 (0.03)	31.11 (0.02)	0.90 (0.01)	511.01 (48.15)	23.49 (0.24)
NaTMA-Y	5.14 (0.01)	2.78 (0.01)	28.89 (0.01)	2.69 (0.01)	12.97 (0.13)	29.45 (0.02)

Figure 7 shows the CO_2_ adsorption isotherms for the three zeolites measured at $288.15\,\textrm{K},298.15\,\textrm{K}$ and $308.15\,\textrm{K}$ up to ${0.12}\,\textrm{bar}$, with the resulting fitted DSL model shown as solid lines. The isotherms are displayed in this pressure range as the maximum partial pressure of CO_2_ attained in the ZLC experiments is ${0.10}\,\textrm{bar}$. The complete isotherms along with the model predictions are shown in Figure S4 in the Supporting Information, and Table 5 gives the fitting parameters along with the associated 95% confidence intervals. The DSL model describes the experimental equilibrium data of CO_2_ on these materials accurately under the conditions studied. Of the three materials, zeolite H-Y exhibits the lowest CO_2_ capacity, as well as the lowest dimensionless Henry’s law constant as shown in Figure S5 in the Supporting Information. Na-Y and NaTMA-Y show similar behavior in terms of non-linearity and adsorption capacity, however, both of these characteristics are weaker for NaTMA-Y as reflected in the dimensionless Henry’s law constants. A detailed analysis of the equilibrium sorption behavior at low and high pressure on these materials was presented in our previous work [27].

### Modeling of the ZLC System

#### Modeling of empty column response

**Fig. 8:**
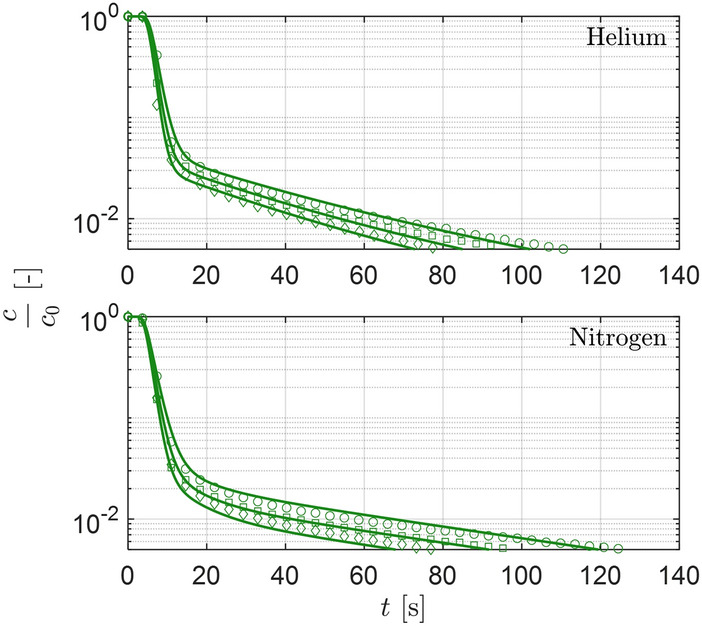
Blank experimental responses using helium and nitrogen as a carrier gas for the ZLC setup obtained at three different inlet inert gas flow rates $F^\textrm{in} = {50}\,\textrm{cm}^{3}\,\textrm{min}^{-1}$**(circles)**, $F^\textrm{in} = {60}\,\textrm{cm}^{3}\,\textrm{min}^{-1}$ (squares) and $F^\textrm{in} = {70}\,\textrm{cm}^{3}\,\textrm{min}^{-1}$ (diamonds) at $y_0 = 0.10$. The solid green line shows the blank model (describing advection, diffusion, and mixing in the experimental setup downstream of the adsorbent particle, Section S1 in the Supporting Information) fitted to the experimental data.

ZLC experiments were first carried out using an empty cell to characterize the empty column response of the setup. This is done by parameterizing a blank model that is then used to predict the full experimental response (composite response of blank and desorption) given a certain desorption response from the adsorbent. Figure 8 shows the experimental blank responses for the ZLC setup along with the corresponding model fits for the individual response curves with an initial feed composition of $y^\textrm{0}_\mathrm {CO_2} = 0.10$. The experiments were carried out at the flow rates at which ZLC desorption experiments were carried out, namely 50, 60, and ${70}\,\textrm{cm}^{3}\,\textrm{min}^{-1}$.

Accurate characterization of the blank response in the system is a key pre-requisite in extracting equilibrium and kinetic parameters from ZLC experiments [25, 43]. To this end, the blank responses for each carrier gas were fitted independently to a model that describes dispersion, mixing, and bulk diffusion within the setup downstream of the adsorbent particle. This was done by modeling the extra volumes as a series of continuous stirred tanks in series with two arrays of a diffusive and mixing volume in parallel connected in series (the blue section of Fig. 1). The capillary tube leading to the detector (MS) and any dynamics within the detector were modeled as multiple tanks in series (the green section of Fig. 1) [25]. The simulated blank responses obtained using the parameters from the fitting of the model to the experiments show a good agreement with the experimental responses within the composition range studied in this work.

The blank model was used to propagate the ZLC response at the outer boundary of the adsorbent particle obtained from solving the ZLC model described in Sect. 3.1 to predict the composite response for the overall system. The derivation of the blank model is described in detail in Section S1 in the Supporting Information.

#### Prediction of CO_2_ sorption kinetics

**Fig. 9:**
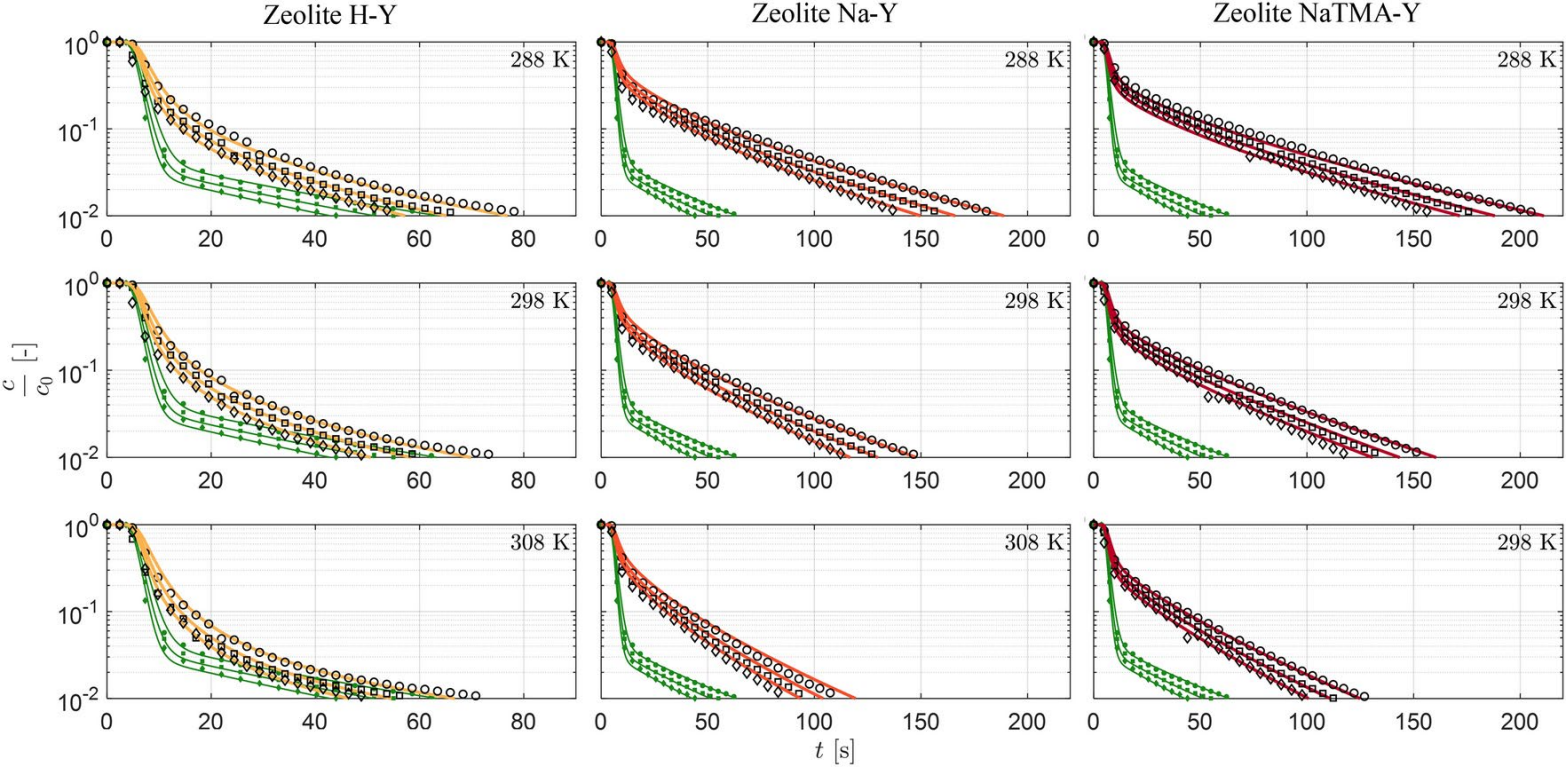
Experimental and simulated desorption responses for Zeolite H-Y, Na-Y, and NaTMA-Y using helium as a carrier gas at ${288.15}\,\textrm{K}$, ${298.15}\,\textrm{K}$, and ${308.15}\,\textrm{K}$, at $F^\textrm{in} = {50}\,\textrm{cm}^{3}\,\textrm{min}^{-1}$ (circles), $F^\textrm{in} = {60}\,\textrm{cm}^{3}\,\textrm{min}^{-1}$ (squares) and $F^\textrm{in} = {70}\,\textrm{cm}^{3}\,\textrm{min}^{-1}$ (diamonds) at $y_0 = 0.10$. The markers represent the time evolution of the experimental CO_2_ concentration at the detector normalized by the initial gas phase concentration, $c_\textrm{0}$. The solid curves indicate the corresponding simulated response generated using the model described in Sect. 3.1 with model parameters given in Table 6. The green symbols and corresponding curves represent the experimental and modeled blank responses of the setup at the respective flow rates.

Parameter estimation was carried out to obtain the optimal values for $\tau _\textrm{mac,PP}$ by fitting the model described in Section 3.1 by imposing macropore diffusion control. For each adsorbent, all 9 experiments using helium as the carrier gas were fitted simultaneously to obtain the optimal value of this parameter. Figure 9 shows time-resolved experimental and model ZLC curves for the three zeolites at ${288.15\,\textrm{K},298.15\,\textrm{K}}$ and $308.15\,\textrm{K}$ at 50, 60, and ${70}\,{\textrm{cm}^{3}\,\textrm{min}^{-1}}$ starting from an initial CO_2_ partial pressure of ${0.10}\,\textrm{bar}$. Qualitatively, we see the expected trends for all three adsorbents, such as shifting of the response to shorter times with increasing flow rate and temperature (lower initial equilibrium loading), and increasing slope of the responses at low compositions with temperature (faster kinetics). We can also see that the Na-Y and NaTMA-Y systems exhibit characteristic long-time asymptotes within the lower limit of detector accuracy ($y=0.001$ or $c/c_0=0.01$). This, for a kinetically controlled experiment, yields information on the macropore diffusivity of the system. As discussed previously, the faster decay of the effluent composition for the H-Y system means a long-time asymptote is not observed within the measurement range. The model predictions using optimal values for the tortuosity match the ZLC responses closely for all three materials at different temperatures and flow rates, particularly in the long-time region of the curves. This is particularly noteworthy considering that the kinetics in these experiments are parameterized by a single unknown parameter ($\tau _\textrm{mac,PP}$), with the rest of the textural and adsorption isotherms being measured independently.

**Table 6:** Summary of kinetic parameters for zeolites H-Y, Na-Y, and NaTMA-Y. Macropore tortuosity ($\tau _\textrm{mac,PP}$) were obtained by fitting the ZLC responses from experiments using CO_2_ using the approach presented in Section 3.4.

Material	$\tau _\textrm{mac,PP}$
	[-]
H-Y	1.70
Na-Y	1.33
NaTMA-Y	2.51

The values obtained for the tortuosity $\tau _\textrm{mac,PP}$ upon parameter estimation are shown in Table 6. First, we note that all values for $\tau _\textrm{mac,PP}$ are all greater than 1. Any value of $\tau _\textrm{mac,PP} < 1$ would not be physically consistent unless external effects that influence the rate of diffusion (*e*.*g*. local constrictions/blockage, and other structural features [44]) are present in addition to the two contributions considered. For a system where the dominant mechanisms for diffusion are Knudsen and molecular diffusion, a value of $\tau _\textrm{mac,PP} = 1$ sets the upper bound for the effective macropore diffusivity, which when applying the parallel pore model gives $D_{\textrm{mac}}^{\textrm{e}}=\varepsilon _\textrm{p} D_{\textrm{p}}$. The values observed for H-Y and Na-Y here ($\tau _\textrm{mac,PP} = 1.70 -1.33$) are lower than those typically reported for shaped FAU zeolites (typically in the range of $2.0-3.0$ [14, 23]. We will elaborate further on this comparison in Sect. 5.3.

## Discussion

### Effective macropore diffusivity

**Fig. 10:**
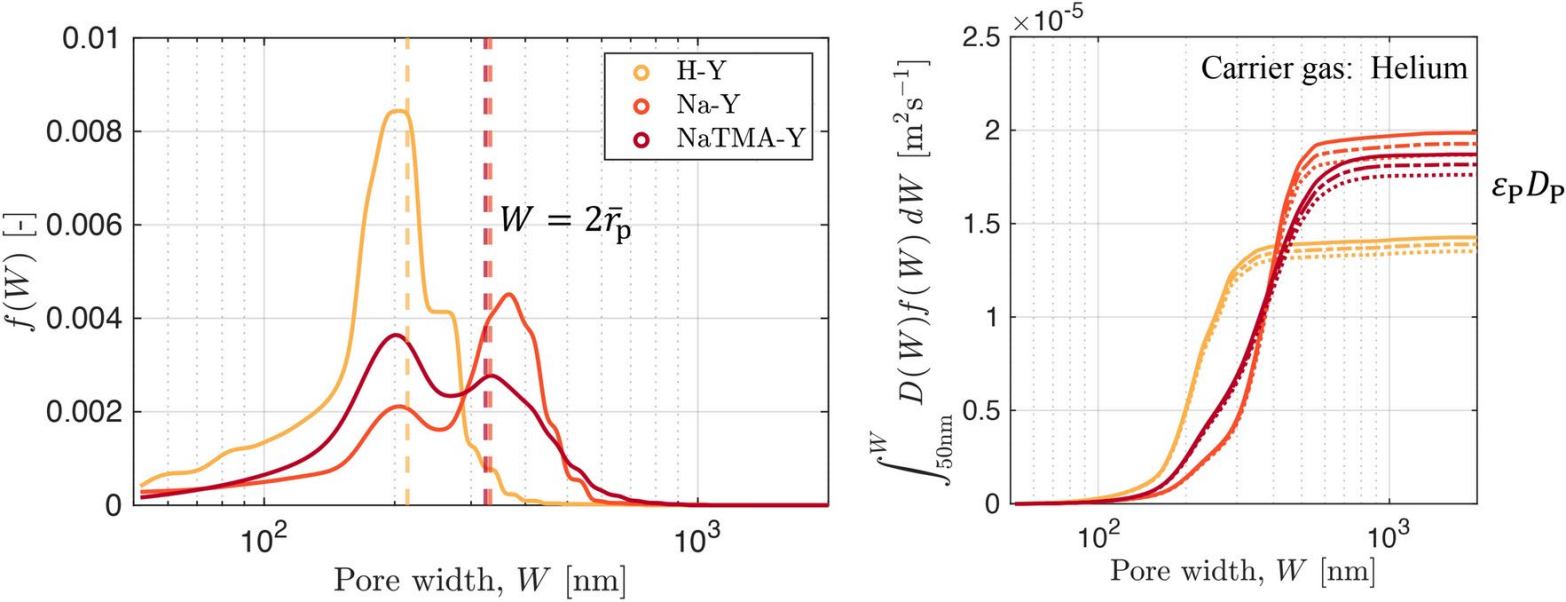
Normalized kernel probability distributions for the macropore PSDs (*f*(*W*)) with the mean pore widths shown as dashed lines for the three adsorbents. The experimental MIP data is removed for visual clarity but is shown in Fig. 6 (left). Cumulative product of *f*(*W*) and *D*(*W*) (the theoretical diffusivity with contributions from Knudsen and molecular diffusivity, shown in Fig. 3) for CO_2_ in helium as a function of pore width, *W*. As given in Eq. 17, the value on the vertical axis of this plots at $W=W_\textrm{max}$ gives $\varepsilon _\textrm{p} D_\textrm{p}$ for the given PSD (right)

Figure 10 shows the fitted probability distributions for the macropore PSDs, *f*(*W*), along with the graphical representation of the parallel pore model (Eq. 17) used to compute the theoretical pore diffusivity ($\varepsilon _\textrm{p} D_{\textrm{p}}$) for each material at the different temperatures. Table 7 reports the values of $\varepsilon _\textrm{p} D_{\textrm{p}}$ for CO_2_ in helium computed using the parallel pore model formulation and the resulting values of the effective macropore diffusivity ($D_{\textrm{mac}}^{\textrm{e}}$, Eq. 16). We can see that the values of $\varepsilon _\textrm{p} D_{\textrm{p}}$ for Na-Y and NaTMA-Y are similar. This is expected as the macropore PSDs for these two materials are very similar (bimodal around similar mean pore widths shown as dashed lines). The two materials differ structurally in terms of the distribution of the two modes of the PSD with Na-Y consisting of a higher proportion of its macroporosity in the wider pore region, as seen in Figure 10. The result is that $\varepsilon _\textrm{p} D_{\textrm{p}}$ for Na-Y is slightly higher than that for NaTMA-Y. H-Y exhibits the lowest value of $\varepsilon _\textrm{p} D_{\textrm{p}}$ when applying the parallel pore model. The macropore PSD for H-Y appears unimodal and exhibits a much higher proportion of its macroporosity in smaller macropores than Na-Y and NaTMA-Y. This results in a greater relative contribution of Knudsen diffusion to the macropore diffusivity than molecular diffusion, and thus a smaller resulting value for $\varepsilon _\textrm{p} D_{\textrm{p}}$.

**Table 7:** Effective macropore diffusivity of CO_2_ in helium computed using the tortuosity factor $\tau _\textrm{mac,PP}$ given in Table 6 by using Eq. 16 along with corresponding values for $\varepsilon _\textrm{p} D_\textrm{p}$ obtained using the parallel pore model (Eq. 17).

Material	$\varepsilon _\textrm{p}$	$\tau _\textrm{mac,PP}$	*T*	$\varepsilon _\textrm{p} D_{\textrm{p}}$(Helium)	$D_{\textrm{mac}}^{\textrm{e}}$(Helium)
	[−]	[ −]	[$$\textrm{K}$$]	[$$\times 10^{-5}$$$$\textrm{m}^{2}\,\textrm{s}^{-1}$$]	[$$\times 10^{-5}$$$$\textrm{m}^{2}\,\textrm{s}^{-1}$$]
			288.15	1.35	0.795
H-Y	0.54	1.70	298.15	1.39	0.817
			308.15	1.43	0.839
			288.15	1.86	1.405
Na-Y	0.47	1.33	298.15	1.93	1.449
			308.15	1.98	1.493
			288.15	1.76	0.702
NaTMA-Y	0.43	2.51	298.15	1.82	0.724
			308.15	1.87	0.746

The obtained values of effective macropore diffusivity ($D_{\textrm{mac}}^{\textrm{e}}$) for Na-Y are larger than those for H-Y and NaTMA-Y (which are both similar), as a result of a lower tortuosity. This is also correlated with the macropore size distributions given in Fig. 10, where Na-Y exhibits a narrower PSD compared to NaTMA-Y (Table 4) and a greater macropore volume compared to NaTMA-Y (raw MIP data in Figure S2 in the Supporting Information). However, as the tortuosity is a single parameter that lumps together several factors that cannot be measured using these experiments, the current results alone cannot definitively relate these textural properties to kinetics.

Hu et al. [23] reported a value of $D_{\textrm{mac}}^{\textrm{e}} = 2.67 \times 10^{-6} \textrm{m}^{2}\,\textrm{s}^{-1}$ at ${311}\,\textrm{K}$ via ZLC for commercial zeolite 13X (Na-X) beads. This is about 5 times smaller than the measured values for Na-Y in this study. Hossain et al. [45] reported a value for the reciprocal time constant for macropore diffusion in zeolite 13X to be $D_{\textrm{mac}}^{\textrm{e}} /{R_\textrm{P}^2} = {3.318}\,\textrm{s}^{-1}$ at ${298.15}\,\textrm{K}$, which is once again smaller than that for Na-Y in this study of ${8.06}\,\textrm{s}^{-1}$ at the same temperature. In addition to being a different parent zeolite (*i*.*e*., 13X being a low silica sodium faujasite), this difference could also be attributed to a distinct shaping procedure used during the production of the pellets resulting in a different macropore structure. Furthermore, the pellets prepared in this study are binderless, as opposed to most commercial 13X pellets which typically have a binder content of $15-25</span>~\%$ by weight [46]. The macroporosity of the pellets in this work ($\varepsilon _\textrm{p}=0.47$ for Na-Y) is larger than values reported for commercial zeolite 13X beads ($\varepsilon _\textrm{p}=0.269)$ [23]. The absence of binder, combined with the high porosity, could potentially increase the external surface area of the active adsorbent crystals that are in contact with the bulk gas in the macropores (reduced pore plugging) and thus may be an important factor in the improved kinetics that we observed in our pellets [47].

**Fig. 11:**
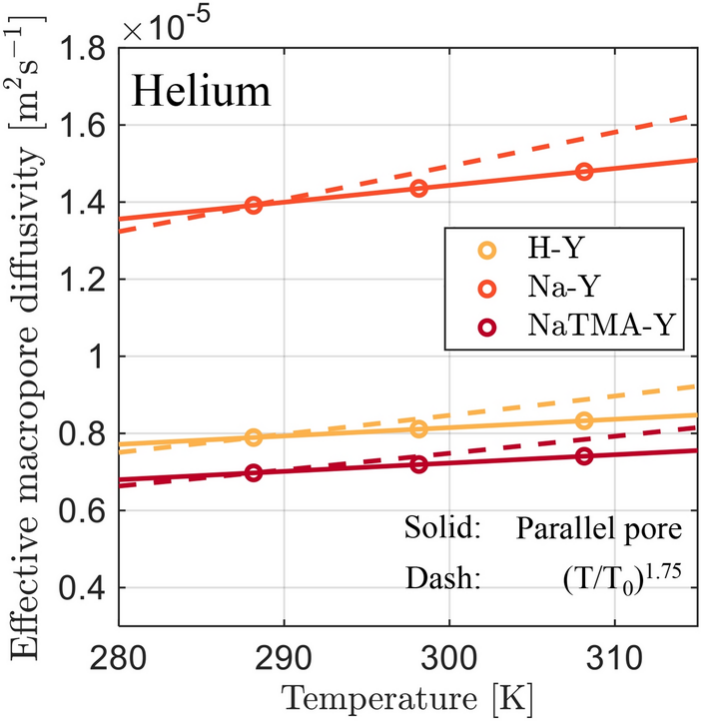
Effective macropore diffusivity of CO_2_ in helium as a function of temperature (empty circles) calculated with Eq. 16 using the optimal values for $\tau _\textrm{mac,PP}$ shown in Table 7. The solid lines correspond to linear fits of the discrete points, and the dashed lines are predictions based on the temperature dependence given in Friedrich et al. [24] in the form $D_{\textrm{mac}}^{\textrm{e}} = D_{\textrm{mac,0}}^{\textrm{e}}(T/288.15)^{1.75}$.

$D_{\textrm{mac}}^{\textrm{e}}$ depends on temperature and the results shown in Fig. 11 indicate a near linear relationship between the effective diffusivity and temperature in the range studied for these materials, which differs from previously reported relationships (*i*.*e*., $D_{\textrm{mac}}^{\textrm{e}} \propto T^{1.75}$ for ZLC experiments on zeolite 13X, dashed lines [24]). In our approach, we explicitly fit for $\tau _\textrm{mac,PP}$ and use expressions from kinetic theory to compute $\varepsilon _\textrm{p} D_{\textrm{p}}$, as opposed to starting with an assumed temperature dependence for $D_{\textrm{mac}}^{\textrm{e}}$. We preferred the former approach as the temperature dependence of $D_{\textrm{mac}}^{\textrm{e}}$ is expected to change depending on the relative contributions of molecular diffusivity ($\propto {T^{1.5}}$) and Knudsen diffusivity ($\propto {T^{0.5}}$) to the overall diffusivity. This will be discussed in more detail in Sect. 5.4.

### Prediction of CO_2_ sorption kinetics in nitrogen

The change of the carrier gas from helium to nitrogen changes the theoretical diffusivity ($\varepsilon _\textrm{p} D_\textrm{p}$) due to the difference in molecular diffusivity of CO_2_ in the two carrier gases (Eq. 20). The results in Table 8 (and Figure S6 in the Supporting Information) show that the values of $\varepsilon _\textrm{p} D_\textrm{p}$ (and $D_{\textrm{mac}}^{\textrm{e}}$ according to Eq. 16 assuming a constant tortuosity) are consistently smaller when nitrogen is the carrier gas as opposed to helium. This observation serves a dual purpose: (i) it supports the conclusion that these systems are under macropore diffusion control and (ii) it can be used to verify the suitability of the parallel pore model for predicting CO_2_ diffusion kinetics in these adsorbents, as discussed next.

**Table 8:** Effective macropore diffusivity of CO_2_ in nitrogen at ${288.15}\,\textrm{K}$ computed using the tortuosity factor $\tau _\textrm{mac,PP}$ given in Table 6 by using Eq. 16 along with corresponding values for $\varepsilon _\textrm{p} D_\textrm{p}$ obtained using the parallel pore model (Equation 17).

Material	$\varepsilon _\textrm{p}$	$\tau _\textrm{mac,PP}$	*T*	$\varepsilon _\textrm{p} D_{\textrm{p}}$(Nitrogen)	$D_{\textrm{mac}}^{\textrm{e}}$(Nitrogen)
	[−]	[−]	[$$\textrm{K}$$]	[$$\times 10^{-5}$$$$\textrm{m}^{2}\,\textrm{s}^{-1}$$]	[$$\times 10^{-5}$$$$\textrm{m}^{2}\,\textrm{s}^{-1}$$]
H-Y	0.54	1.70	288.15	0.94	0.555
Na-Y	0.47	1.33		1.17	0.882
NaTMA-Y	0.43	2.51		1.12	0.448

**Fig. 12:**
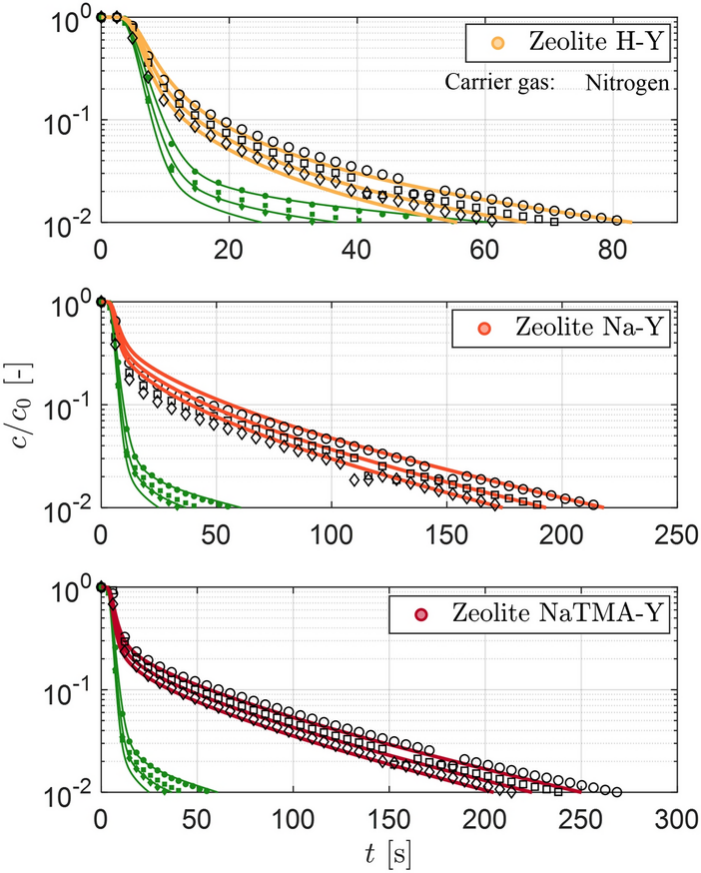
Experimental and predicted desorption responses for the three adsorbents using nitrogen as a carrier gas at ${288.15}\textrm{K}$ at $F^\textrm{in} = {50}\,\textrm{cm}^{3}\,\textrm{min}^{-1}$**(circles)**, $F^\textrm{in} = {60}\,\textrm{cm}^{3}\,\textrm{min}^{-1}$ (squares) and $F^\textrm{in} = {70}\,\textrm{cm}^{3}\,\textrm{min}^{-1}$ (diamonds) at $y_0 = 0.10$. The markers represent the time evolution of the experimental CO_2_ concentration at the detector normalized by the initial gas phase concentration, $c_\textrm{0}$. The solid curves indicate the corresponding simulated response generated using the model described in Sect.3.1 with model parameters given in Table 6. The green symbols and corresponding curves represent the experimental and modeled blank responses of the setup at the respective flow rates.

The theoretical diffusivities $\varepsilon _\textrm{p} D_\textrm{p}$ calculated for CO_2_ in nitrogen (given in Table 8) were used to predict the composite ZLC responses that would be obtained with nitrogen as a carrier gas. Figure 12 shows the experimental ZLC responses using nitrogen as a carrier gas for the three zeolites in carrier gas flowrates of 50, 60 and ${70}\,\textrm{cm}^{3}\,\textrm{min}^{-1}$ at ${288.15}\,\textrm{K}$ along with the model predicted ZLC responses obtained using the $\tau _\textrm{mac,PP}$ obtained from helium experiments. The model prediction is in good agreement with the experimental data, particularly with respect to the slope at low compositions. Additionally, the ratio of the diffusivity of CO_2_ in helium (Table 7) to that in nitrogen (Table 8) for H-Y, Na-Y, and NaTMA-Y were found to be 1.43, 1.59, and 1.57 respectively. For Na-Y and NaTMA-Y, which exhibit the characteristic long-time asymptotes, this is in good agreement with the ratio of the slope of the long-time asymptotes of the composite ZLC response, *i*.*e*., 1.56 and 1.66 respectively.

The findings in this section confirm that: (1) the mass transport in the system is limited by macropore diffusion, as the overall particle diffusivity (as a consequence of macropore diffusion) changes with carrier gas, and (2) for these macropore diffusion-limited systems, the parallel pore model can be used to accurately describe the kinetics of CO_2_ desorption in the presence of different inert gases based on a single fitted parameter, given enough information on the equilibrium and textural properties of the material.

### Single-vs. parallel-pore model formulation

The theoretical diffusivities predicted by the parallel pore model represent cumulative distributions, meaning that the value of $\varepsilon _\textrm{p} D_\textrm{p}$ reported in Table 7 corresponds to the value of the curve at $W=W_\textrm{max}$, *i*.*e*., upon performing the integration of the probability distribution over the whole range of macropore sizes. Accordingly, one can also estimate a value of $\varepsilon _\textrm{p} D_\textrm{p}$ using a characteristic pore width, *i*.*e*., $W=2\overline{r}_\textrm{p}$, the mean pore diameter (given in Table 4), as proposed by Satterfield [39]. In this formulation, $\varepsilon _\textrm{p} D_\textrm{p}$ can be given as follows.

27 $$\begin{aligned} \varepsilon _\textrm{p} D_\textrm{p} = \varepsilon _\textrm{p} \left( \frac{1}{D_{12}^{\textrm{m}}}+\frac{1}{D_j^{\textrm{K}}(2\overline{r}_\textrm{p})}\right) ^{-1} \end{aligned}$$

Using Eq. 27, yields values for $\varepsilon _\textrm{p} D_\textrm{p}$ that are consistently smaller than those obtained using the parallel pore model [34]. Consequently, one expects that to fit the ZLC response curves (*i*.*e*., to yield the same effective macropore diffusivity) the value of the tortuosity obtained using this model must be reduced ($\tau _\textrm{mac,mean}<\tau _\textrm{mac,PP}$). To confirm this, the parameter estimation was repeated using this formulation as described in Sect. 3.4 by assuming the macropore network is characterized by the mean pore diameter ($2\overline{r}_\textrm{p}$).

**Fig. 13:**
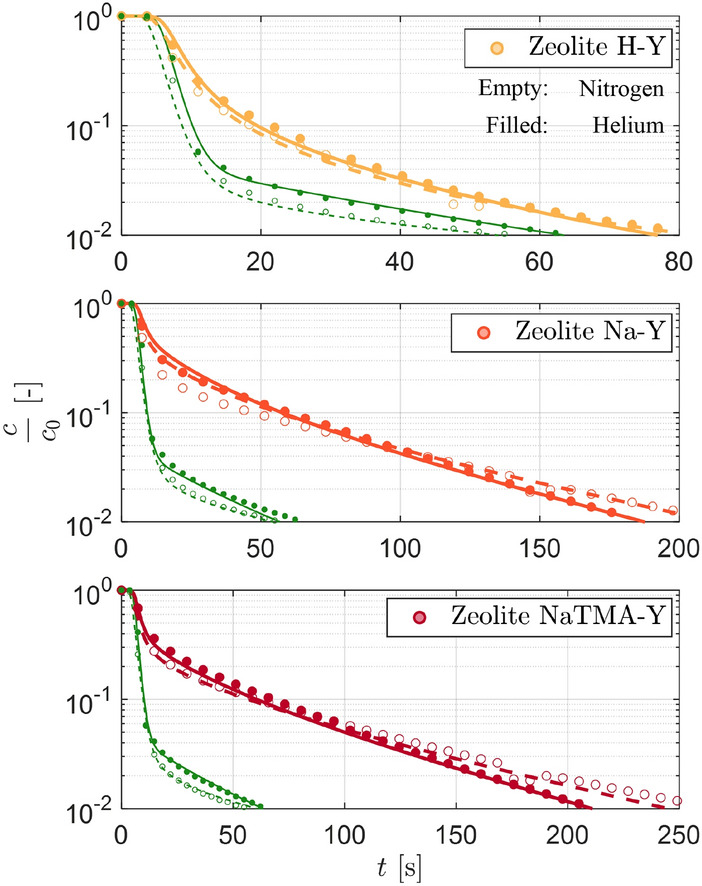
Experimental and predicted desorption responses for the three adsorbents using helium (filled and solid) nitrogen (empty and dashed) as a carrier gas at ${288.15}\,\textrm{K}$ and $F^\textrm{in} = {50}\,\textrm{cm}^{3}\,\textrm{min}^{-1}$ by using the kinetic model given by Eq. 27. The ZLC responses using helium were fitted to obtain $\tau _\textrm{mac,mean}$, and the model was used to predict the response for nitrogen.

Figure 13 shows the fitted (for helium) and predicted (for nitrogen) ZLC responses using this model formulation compared with the experimental responses at ${288.15}\,\textrm{K}$ and $F^\textrm{in} = {50}\,\textrm{cm}^{3}\,\textrm{min}^{-1}$ by using the kinetic model given by Eq. 27. The quality of the fits to the CO$_2$ data and of the description of the N$_2$ data is similar to the one achieved upon using the parallel pore formulation. The resulting values for the effective macropore diffusivity are also similar (to two significant figures) between the two model formulations. However, the values for tortuosity for this formulation ($\tau _\textrm{mac,mean}$) given in Table 9 are unrealistically low ($< 1$) and are therefore a mere result of a fitting exercise, rather than an accurate representation of the system under study. This reduction in tortuosity was also highlighted by Wang and Smith [34] in proposing the parallel pore model, where it was also noted that using Eq. 27 for materials with wide pore size distributions resulted in an apparent temperature dependence of the tortuosity [34]. Since tortuosity is an inherent physical property of the pore structure, it should not exhibit a temperature dependence when calculated with an appropriate model [48]. However, such a dependence was not observed in the narrow temperature range studied in this work. Regardless, the current findings indicate that using a single characteristic length scale in computing $D_\textrm{p}$ is inappropriate for these systems.

**Table 9:** Comparison of tortuosity factors obtained from fitting the ZLC responses from experiments using helium as a carrier gas using the kinetic model given by Eq. 27 ($\tau _\textrm{mac,mean}$), the parallel pore model ($\tau _\textrm{mac,PP}$) and extracted from MIP data ($\tau _\textrm{MIP}$).

Material	$\tau _\textrm{mac,mean}$	$\tau _\textrm{mac,PP}$	$\tau _\textrm{MIP}$
	[-]	[-]	[-]
H-Y	0.56	1.70	1.62
Na-Y	0.84	1.33	1.65
NaTMA-Y	0.98	2.51	1.71

The tortuosity obtained using the two diffusion models can be compared directly as they both correspond to ratios of diffusivity within the macropore network. However, the pore network tortuosity obtained from MIP ($\tau _\textrm{MIP}$) corresponds to a ratio between physical length scales within the pore structure, assuming all pores are straight and cylindrical. This is understood to be a fixed property of the material. Hence, comparing $\tau _\textrm{MIP}$ with the parameters obtained from applying each diffusion model to the ZLC data is a way to support the choice of model. From the results in Table 9 we can conclude that the tortuosity values obtained from applying the parallel pore model are closer to $\tau _\textrm{MIP}$ for all three materials, which themselves are smaller than what is reported in the literature for commercial FAU zeolites.

### Temperature dependence of the effective macropore diffusivity

**Fig. 14:**
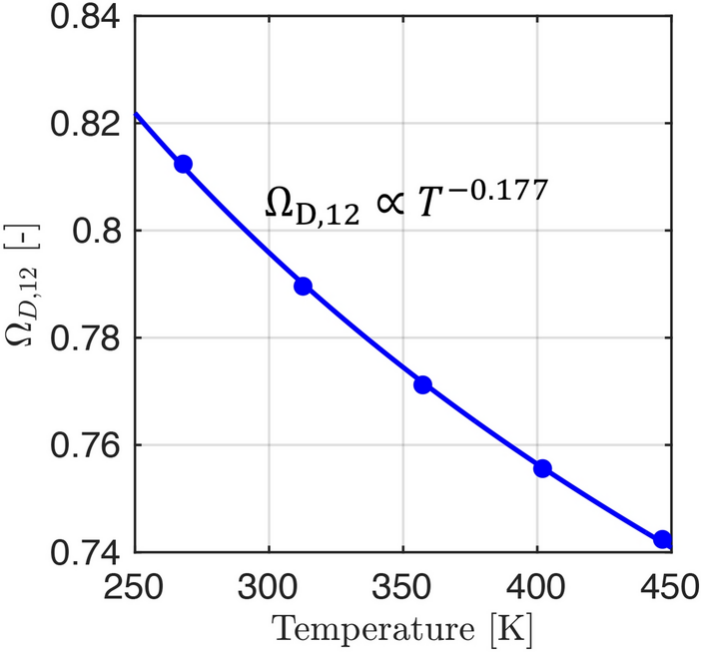
Collision integrals for CO_2_ in helium obtained from tabulated data in Bird et al. [38] (circles), fitted to a polynomial (line) of order $-0.177$.

In this final section, we derive the temperature dependence of the theoretical diffusivity, $D_\textrm{p}$, by using standard kinetic models coupled to either the single- and parallel-pore model formulation. Specifically, we will explore the suitability of a previously adopted power-law formulation [24] and the expected range of the power-law exponent, *b*, namely

28 $$\begin{aligned} D_\textrm{p} = D_\textrm{p,0}\left( \frac{T}{T_0}\right) ^b \end{aligned}$$

where $D_\textrm{p,0}$ is the value of $D_\textrm{p}$ evaluated at $T_0$. The foundational equation used to compute the theoretical diffusivity in these systems is the Bosanquet equation [36] (Eq. 18) which combines contributions from Knudsen and molecular diffusion as two resistances in series. Knudsen diffusivity $D_j^{\textrm{K}}(W)$ (Eq. 19) has a temperature dependence of $D_j^{\textrm{K}}(W) \propto T^{0.5}$. Molecular diffusion $D_{12}^{\textrm{m}}$ (Eq. 20), on the other hand, has a direct temperature dependence of $D_{12}^{\textrm{m}} \propto T^{1.5}$, as well as an indirect temperature dependence due to the temperature-dependent collision integral $\Omega _{D, 12}$. For the temperature range 250 to ${450}\,\textrm{K}$, $\Omega _{D, 12} \propto T^{-0.177}$ as shown in Fig. 14. Therefore, the overall temperature dependence for the molecular diffusivity in this temperature range can be approximated as $D_{12}^{\textrm{m}} \propto T^{1.677}$. This aligns with the approximation of 1.7 for the same exponent given by Ruthven [49].

The Bosanquet equation can thus be rewritten and recasted to the form,

29 $$\begin{aligned} D(W) \approx \left( \frac{a_1a_2WT^{2.177}}{a_1T^{1.677}+a_2WT^{0.5}}\right) \end{aligned}$$

where $a_1$ and $a_2$ are the constant parameters in the expressions for molecular and Knudsen diffusivity respectively. When *W* is very small, the molecular diffusivity ($a_1T^{1.677}$) dominates the denominator and thus $D(W)\propto T^{0.5}$ (Knudsen diffusion dominates). Accordingly, when *W* is very large, Knudsen diffusivity ($a_2WT^{0.5}$) dominates the denominator, and *D*(*W*) is independent of *W*. In this case, $D(W)\propto T^{1.677}$ (bulk diffusion dominates). It follows that the exponent describing the temperature dependence of *D*(*W*) must be bound between 0.5 and 1.677 in this temperature range. Clearly, when both contributions are similar in magnitude, determining an exponent for the temperature dependence is not straightforward and has to be evaluated for each material.

#### Mean pore radius model

**Fig. 15:**
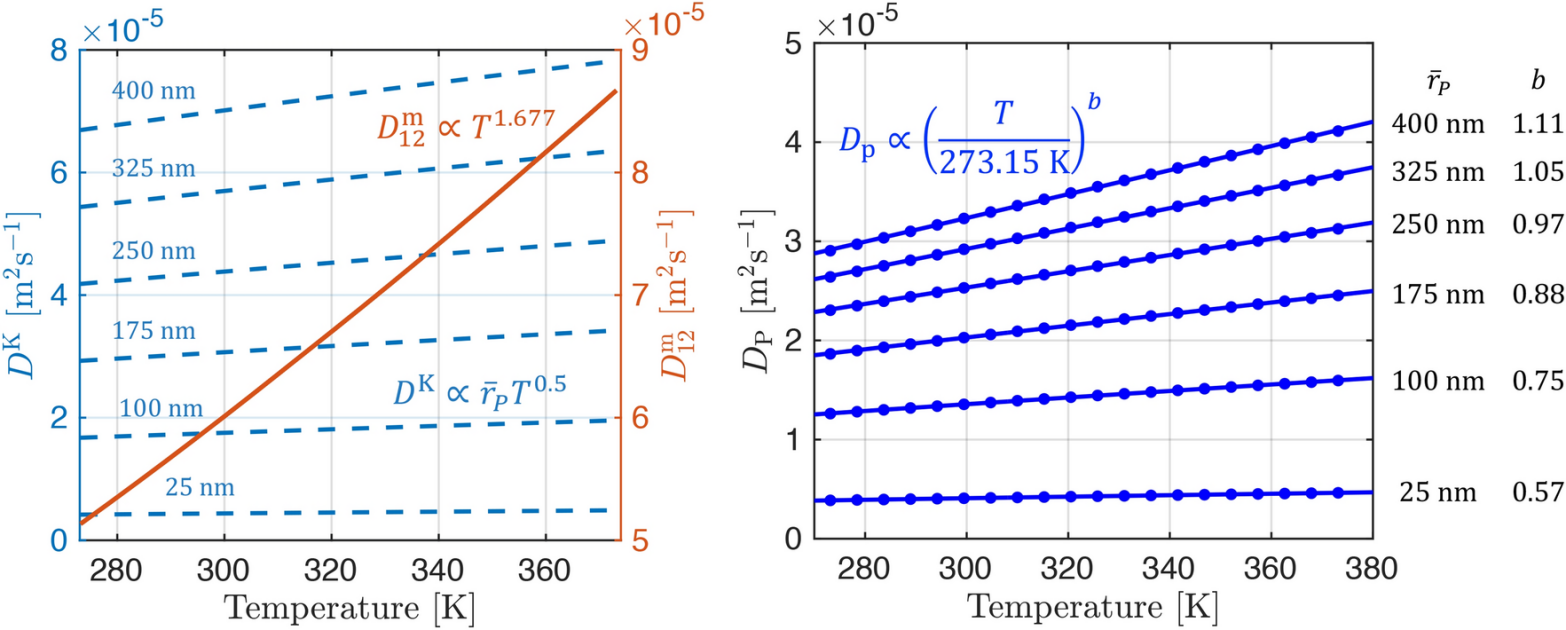
**(left)** Temperature dependence of the individual contributions to the overall theoretical diffusivity from Knudsen $D^{\textrm{K}}$ (blue dashed lines, evaluated using different mean pore radii) and molecular diffusion $D_{12}^{\textrm{m}}$ (red solid line). The Knudsen diffusivity is proportional to the mean pore radius ($\overline{r}_\textrm{p}$) and $T^{0.5}$, while molecular diffusivity is approximately proportional to $T^{1.677}$. **(right)** The theoretical diffusivity $D_{\textrm{p}}$ (blue circles) computed assuming a range of mean pore radii (${25}\,\textrm{nm}$ to ${400}\,\textrm{nm}$) as a function of temperature. The $\overline{r}_\textrm{p}$ values on the right correspond to the mean pore radii evaluated and *b* corresponds to the exponent of the power–law function, Eq. 28, fitted to each case using a reference temperature $T_0$ chosen to be ${273}\,\textrm{K}$.

The left panel in Fig. 15 shows the temperature dependence of Knudsen diffusion (Eq. 19) and molecular diffusion (Eq. 20) of CO_2_ in helium for generic porous materials with mean pore radii ranging from ${25}\,\textrm{nm}$ to ${400}\,\textrm{nm}$ for the range ${273}\,\textrm{K}$ to ${373}\,\textrm{K}$. The right panel shows the corresponding theoretical pore diffusivity of CO_2_ in helium calculated by combining these two contributions using Eq. 27. In the plot, the lines correspond to fits of Eq. 28 with $T_0 =$ 273 K) to the discrete data points shown as blue circles. As expected, the temperature dependence is strongly influenced by the mean pore radius, as it determines the relative contributions of each mechanism when they are both in the same order of magnitude. The exponent *b* ranges from 0.55, at the lower limit for macropores (${50}\,\textrm{nm}$), to 1.11, for a mean pore radius of ${400}\,\textrm{nm}$. We note that the upper limit for this exponent should be 1.677 when molecular diffusion is the controlling mechanism.

#### Parallel pore model

**Fig. 16:**
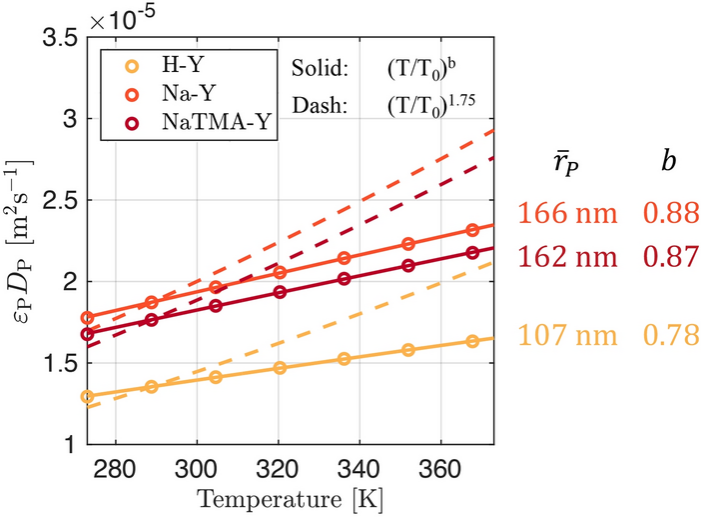
The theoretical pore diffusivity $\varepsilon _\textrm{p} D_{\textrm{p}}$ as a function of temperature predicted using the parallel pore model (circles) with the power law function Eq. 28 with the reference temperature $T_0$ chosen to be ${283}\,\textrm{K}$ shown in solid lines, and using the previously reported exponent of 1.75 (dashed lines) for the temperature range ${273}\,\textrm{K}$ to ${373}\,\textrm{K}$.

The above analysis is extended here to the parallel pore model formulation by using the PSDs of the three zeolite adsorbents. The results are shown in Fig. 16 for the temperature range ${273}\,\textrm{K}$ to ${373}\,\textrm{K}$, along with fitted power-law function, Eq. 28, with $T_0 =$ 283 K. We observe that for the three adsorbents the value of the exponent *b* varies in the range 0.78–0.88. As observed for the single-pore model, the value of *b* is correlated with the values of the mean pore radii of the adsorbents. We note that for the experiments conducted here, the temperature dependence of $\varepsilon _\textrm{p} D_{\textrm{p}}$ can be described with sufficient accuracy by a linear relationship (Fig. 11), this being the result of the limited temperature range covered in the experiments (288 to ${308}\textrm{K}$). Importantly, we observe that, upon extrapolation, the value of $b=1.75$ proposed in previous studies could lead to substantial differences in the predicted values of $\varepsilon _\textrm{p} D_{\textrm{p}}$.

## Conclusions

In this work, we evaluated the kinetics of CO_2_ sorption on binderless pellets of three Y-type zeolites, namely H-Y, Na-Y, and NaTMA-Y, using the Zero-Length Column (ZLC) technique. The binderless pellets of the three zeolites were thoroughly characterized to provide the necessary parameters for the analysis of the ZLC experiments, namely the equilibrium isotherms for CO_2_, macropore size distributions, crystal density, and porosity. The ZLC experiments were carried out using the CO$_2$-helium system at 288.15, 298.15 and ${308.15}\,\textrm{K}$ using three inert gas flow rates: 50, 60, and ${70}\,\textrm{cm}^3 \,\textrm{min}^{-1}$. ZLC experiments carried out at ${288.15}\,\textrm{K}$ using nitrogen as the carrier gas at the same three gas flow rates confirmed that the three zeolite systems were under kinetic control and macropore diffusion control. The 1-D Fickian radial diffusion model was applied to describe the experimental ZLC response curves by using the macropore tortuosity as the sole fitting parameter for each Y-type zeolite. In this endeavour, we only used the measurements for the CO$_2$-helium system and applied the parameterized model to predict the experiments with the CO$_2$-nitrogen system. The accurate description of the two sets of experiments confirms that the macropore tortuosity is independent of the carrier gas. Importantly, our results indicate that the use of a single characteristic mean pore size to compute the theoretical pore diffusivity is inappropriate, as it yields tortuosity values below the physical limit of 1. Rather, one should account for the whole range of macropore sizes encountered in binded pellets-an aspect that in our study was achieved through the so-called parallel-pore model formalism. We also show that for low pressure open flow systems the temperature dependence of the macropore diffusivity follows a power-law function with an exponent that must be bound between 0.5 and 1.7. This range is the result of the relative contributions of Knudsen and molecular diffusion to mass transport. Our result fall within this range of values, showing a nearly linear increase of the effective macropore diffusivity with temperature.

## Electronic supplementary material

Below is the link to the electronic supplementary material.

Supplementary file1
