# Supplementary material for: Effective macropore diffusivity of carbon dioxide on binderless pellets of Y-type zeolites
Source: Adsorption (Boston). 2025 Jan 31;31(2):39. doi: 10.1007/s10450-025-00599-3 (PMC11785662; doi:10.1007/s10450-025-00599-3)
Supplement: Supplementary file 1 — Supplementary file1 [file 10450_2025_599_MOESM1_ESM.pdf]

Supporting Information (SI) for "Effective macropore diffusivity of carbon dioxide on binderless pellets of Y-type zeolites"

Hassan Azzan,[†] Killian Gmyrek,[†] David Danaci,[†,‡,¶] Ashwin Kumar Rajagopalan,[§]
Camille Petit,[†,*] Ronny Pini[†,*]

Contents

S1 Modelling extra column volumes **2**

S2 Mercury intrusion porosimetry (MIP) **4**

S3 Nitrogen sorption at 77 K **5**

S4 Parameters for calculating molecular diffusivity **6**

S5 Effective pore diffusivity for macropore limited systems **7**

S6 CO_2 equilibrium isotherms and DSL fits **8**

S7 Graphical representation of parallel pore model **9**

[*] Email: camille.petit@imperial.ac.uk, rpini@imperial.ac.uk
[†] Department of Chemical Engineering, Imperial College London, London, SW7 2AZ, United Kingdom
[‡] The Sargent Centre for Process Systems Engineering, Imperial College London, London, SW7 2AZ, United Kingdom
[¶] I-X Centre for AI in Science, Imperial College London, London, W12 0BZ, United Kingdom
[§] Department of Chemical Engineering, The University of Manchester, Manchester, M13 9PL, United Kingdom

S1 Modelling extra column volumes

We first presented a version of this model in our previous work on the simultaneous estimation of adsorption equilibria and kinetics on shaped adsorbents[1]. We model the blank volume (*Segment I* and *Segment II*) in the experimental setup shown in Figure S1 of the main text (blue and green) by modeling individual components of the setup that are lumped into sections that exhibit a given flow behavior.[2–5]

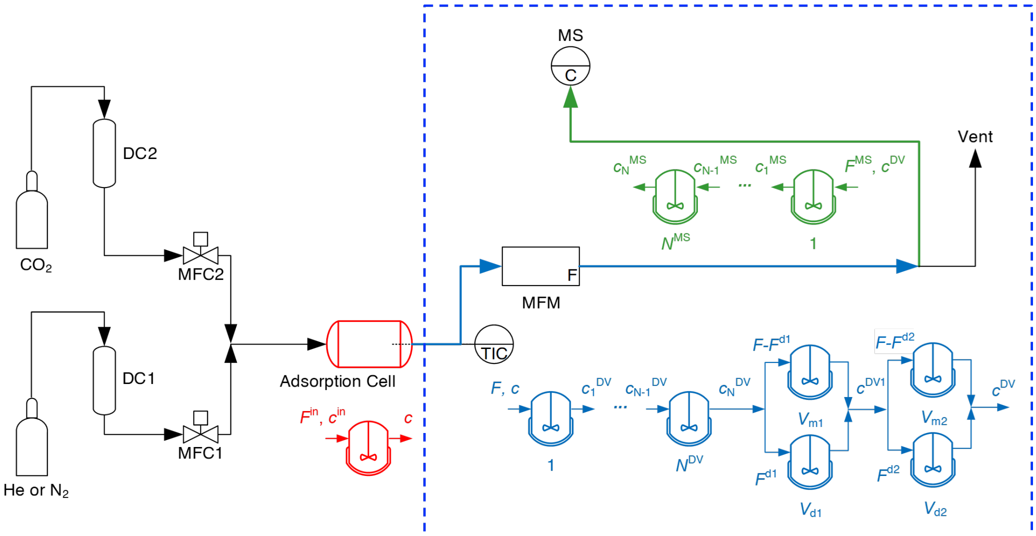

Figure S1: Schematic of the experimental setup used for the blank ZLC experiments. The region in highlighted with a blue dashed box corresponds to the volume described by the blank model.

Segment I: The entire segment, shown in blue in Figure 1 of the main text, has the same gas flow rate $F(t)$ as the outlet of the CSTR that models the gas adsorption in the adsorbent. We described the gas flow in the volume that exhibits a plug flow behavior with axial dispersion using a series of N^{DV} CSTRs, with a total volume V^{DV} [m^3]. Note that the higher the number of CSTRs N^{DV}, the lower the axial dispersion. The mass balance around a CSTR p is given as

$$\frac{\mathrm{d}c_p^{\text{DV}}}{\mathrm{d}t} = \frac{F(t)}{V^{\text{DV}}/N^{\text{DV}}}\left(c_{p-1}^{\text{DV}} - c_p^{\text{DV}}\right) \qquad p = 1, \ldots, N^{\text{DV}} \tag{S1}$$

where c_p^{S} [mol m^{-3}] is the concentration of the gas at the outlet of CSTR p and $F(t)$ is the volumetric mixture gas flow rate at the outlet of the CSTR that models the gas sorption in the adsorbent, obtained by solving the system of equations given by Equation 7 of the main text.

We described the gas flow in the volume that exhibits a diffusive and mixing behavior as two arrays of two CSTRs in parallel, which are connected in series. This was necessary to accurately describe the blank response at low CO_2 compositions close to the new detector threshold of 0.1%. The first array is characterized by a mixing volume V^{m1} [m^3] with a volumetric mixture gas flow rate $\left(F(t) - F^{\text{d1}}\right)$ [m^3 s^{-1}] and a diffusive volume V^{d1} [m^3] with a constant volumetric mixture gas flow rate F^{d1} [m^3 s^{-1}]. The mass balance around these two reactors is given by

$$\begin{aligned} \frac{\mathrm{d}c^{\text{m1}}}{\mathrm{d}t} &= \frac{\left(F(t) - F^{\text{d1}}\right)}{V^{\text{m1}}}\left(c_{N^{\text{DV}}}^{\text{DV}} - c^{\text{m1}}\right) \\ \frac{\mathrm{d}c^{\text{d1}}}{\mathrm{d}t} &= \frac{F^{\text{d1}}}{V^{\text{d1}}}\left(c_{N^{\text{DV}}}^{\text{DV}} - c^{\text{d1}}\right) \end{aligned} \tag{S2}$$

where c^{m1} [mol m^{-3}] and c^{d1} [mol m^{-3}] are the concentrations of the gas at outlet of the first array of mixing and diffusive volumes, respectively, and $c^{\mathrm{DV}}_{N^{\mathrm{DV}}}$ [mol m^{-3}] is the concentration at the outlet of CSTR N^{DV}, obtained from Equation S1.

Next, we combine the gas from the mixing and the diffusive volume as

$$c^{\mathrm{DV1}} = \frac{\left(F(t) - F^{\mathrm{d1}}\right) c^{\mathrm{m1}} + F^{\mathrm{d1}} c^{\mathrm{d1}}}{F(t)} \tag{S3}$$

which is the inlet to the second array of mixing and diffusive volumes. This is modeled the same as the first array with different magnitudes of mixing (V^{m2}) and diffusive (V^{d2}) volumes and flowrate into the diffusive volume (F^{d2}). The concentration at the outlet from this section (c^{DV}) is the concentration of gas exiting *Segment I*, with a gas concentration c^{DV} [mol m^{-3}] defined as follows.

$$c^{\mathrm{DV}} = \frac{\left(F(t) - F^{\mathrm{d2}}\right) c^{\mathrm{m2}} + F^{\mathrm{d2}} c^{\mathrm{d2}}}{F(t)} \tag{S4}$$

Note that we assumed the pressure and temperature of the gas from the two volumes to be identical. The initial conditions for Equations S1 and S2 are

$$\begin{aligned}
c_p^{\mathrm{DV}}(0) &= \frac{Py^0}{RT} \qquad p = 1, \ldots, N^{\mathrm{DV}} \\
c^{\mathrm{m1}}(0) &= \frac{Py^0}{RT} \\
c^{\mathrm{d1}}(0) &= \frac{Py^0}{RT} \\
c^{\mathrm{m2}}(0) &= \frac{Py^0}{RT} \\
c^{\mathrm{d2}}(0) &= \frac{Py^0}{RT}
\end{aligned} \tag{S5}$$

where P [Pa] is the pressure, T [K] is the temperature and y^0 [–] is the initial gas phase mole fraction in the blank volume of the setup.

Segment II: The entire segment, shown in green in Figure 1 of the main text, has a constant gas flow rate of F^{MS} [m^3 s^{-1}] and it is dictated by the vacuum pump of the MS. We described the gas flow using a series of N^{MS} CSTRs, with a total volume V^{MS} [m^3]. The mass balance around a CSTR q is given as

$$\frac{\mathrm{d}c_q^{\mathrm{MS}}}{\mathrm{d}t} = \frac{F^{\mathrm{MS}}}{V^{\mathrm{MS}}/N^{\mathrm{MS}}} \left(c_{q-1}^{\mathrm{MS}} - c_q^{\mathrm{MS}}\right) \qquad q = 1, \ldots, N^{\mathrm{MS}} \tag{S6}$$

where c_q^{MS} [mol m^{-3}] is concentration of the gas at outlet of CSTR q and F^{MS} [m^3 s^{-1}] is a constant volumetric mixture gas flow rate through the MS, which is independent of the volumetric mixture gas flow rate $F(t)$ in *Segment I.* The initial condition for Equation S6 is

$$c_q^{\mathrm{MS}}(0) = \frac{Py^0}{RT} \qquad q = 1, \ldots, N^{\mathrm{MS}} \tag{S7}$$

where P [Pa] is the pressure, T [K] is the temperature and y^0 [–] is the initial gas phase mole fraction in the blank volume of the MS.

S2 Mercury intrusion porosimetry (MIP)

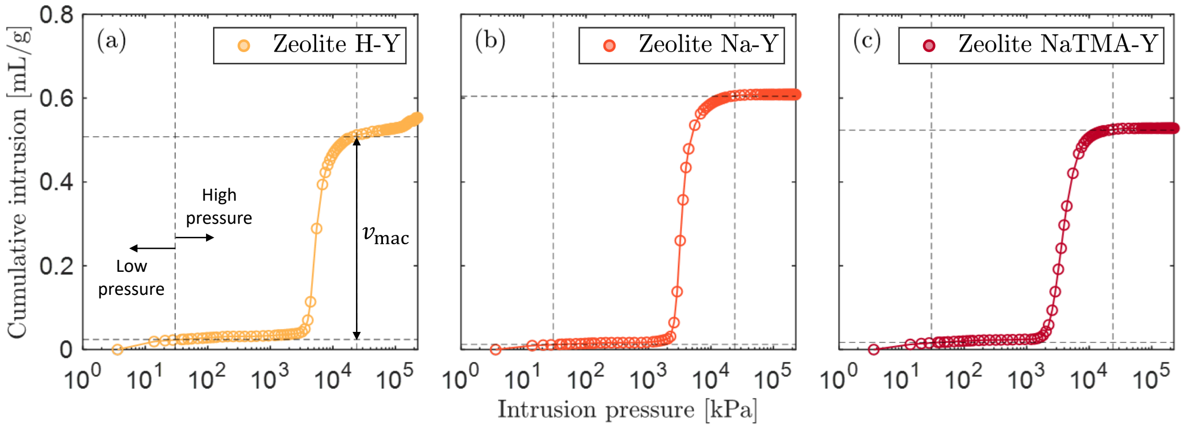

Figure S2: Mercury intrusion porosimetry results for H-Y (yellow), Na-Y (orange), and NaTMA-Y (maroon) shown as cumulative mercury intrusion as a function of pressure obtained at low (up to 30 kPa) and high pressure (from 30 kPa to 227,490 kPa). The low pressure data is used to obtain the envelope volume of the adsorbents v_p. The macropore volume v_mac is the difference in cumulative intrusion between 3 and 24,060 kPa which corresponds to the pressure at which the intruded pore diameter is 50 nm.

S3 Nitrogen sorption at 77 K

All three zeolites exhibit a relatively large proportion of micropore volumes (31%, 36%, and 39% of total pore volume for H-Y, Na-Y, and NaTMA-Y respectively) as is expected from faujasites. Although NaTMA-Y is produced from the parent Na-Y, it exhibits a lower micropore volume after cation exchange and activation, whilst H-Y shows the lowest micropore volume as summarised in Table 4 of the main text. The volume of mesopores is relatively small for all samples (9%, 3%, and 2% of total pore volume for H-Y, Na-Y, and NaTMA-Y respectively), with H-Y showing the largest value. S_{MIP} corresponds to the macropore surface area obtained from MIP measurements and S_{BET} is equivalent to the combination of "*micropore capacity*" and the "*monolayer content*" of the external surface of the crystals[6] and is obtained by applying the BET equation to the N_2 isotherm at 77 K. By considering the results from N_2 isotherms at 77 K and MIP, we see that the S_{BET} is correlated to the micropore volume as expected, while S_{MIP} is correlated to locations of the peaks in the macropore size distribution (i.e., the mean pore radius $\bar{r}_{\mathrm{p}}$).

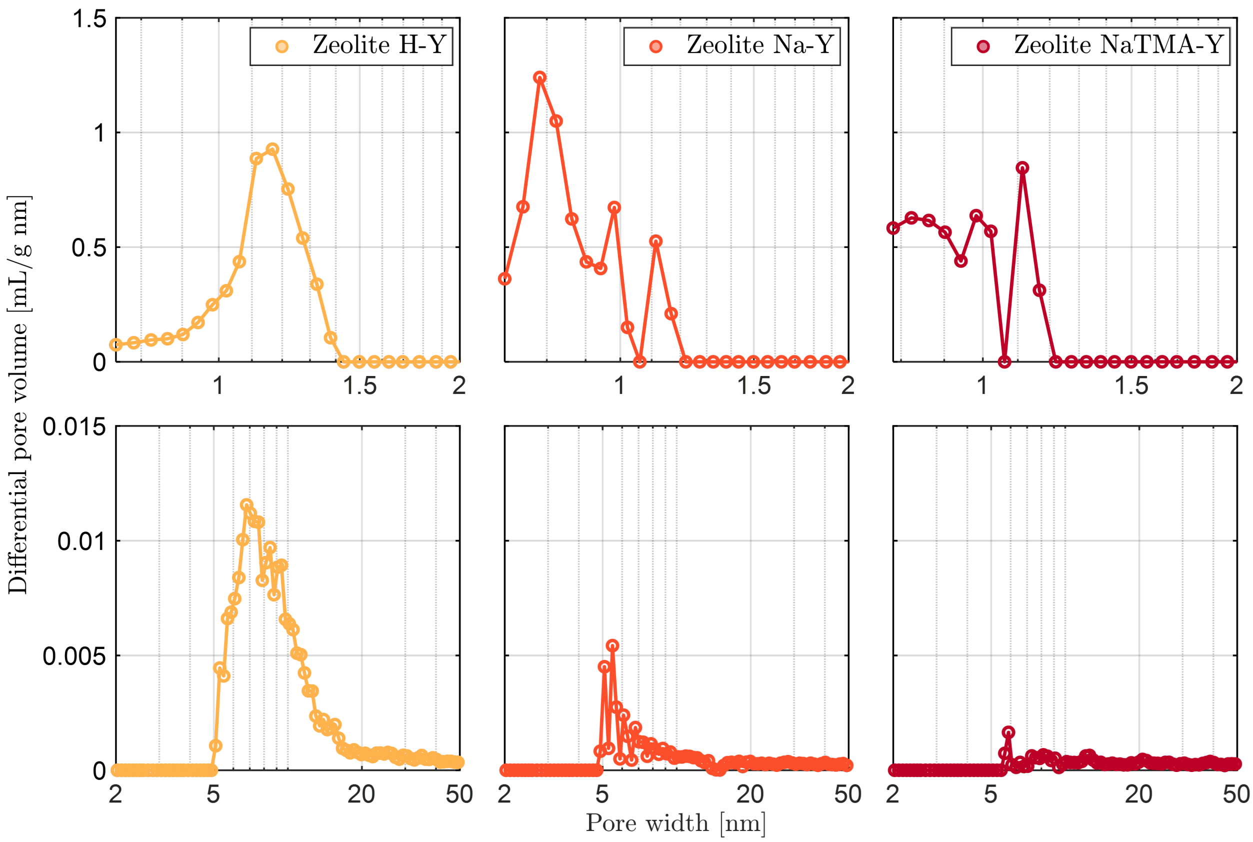

Figure S3: Pore size distribution in the micropore **(top)** and mesopore **(bottom)** regions for H-Y (yellow), Na-Y (orange), and NaTMA-Y (maroon) obtained by low-pressure volumetric nitrogen isotherms measured at 77 K. The solid lines connect the discrete data points as a visual guide.

S4 Parameters for calculating molecular diffusivity

This section provides the parameters used to compute the molecular diffusivity assuming steady-state equimolar counter diffusion of a binary mixture of CO_2 in each carrier gas using the Chapman-Enskog equation (Equation 19). These values were computed from tabulated values by Bird *et al.*[7]. For computing the temperature dependence of the molecular diffusivity (and thus the theoretical pore diffusivity) for each material the required parameter values were obtained by linear interpolation of tabulated data.

Table S1: Parameters used for calculating molecular diffusivity assuming steady-state equimolar counter diffusion of a binary mixture of CO_2 in the carrier gas (He) using the Chapman-Enskog equation.

**Parameter**	$\sigma_{\mathrm{CO_2/He}}$ [Å]	$\Omega_{D,\mathrm{CO_2/He}}$ [-]		
Temperature [K]	-	288.15	298.15	308.15
Value	3.25	0.8021	0.7969	0.7919

Table S2: Parameters used for calculating molecular diffusivity assuming steady-state equimolar counter diffusion of a binary mixture of CO_2 in the carrier gas (N_2) using the Chapman-Enskog equation.

**Parameter**	$\sigma_{\mathrm{CO_2/N_2}}$ [Å]	$\Omega_{D,\mathrm{CO_2/N_2}}$ [-]		
Temperature [K]	-	288.15	298.15	308.15
Value	3.87	1.0069	0.9966	0.9867

S5 Effective pore diffusivity for macropore limited systems

$D_{\mathrm{p}}^{\mathrm{e}}$ is the effective pore diffusivity which can be obtained by rearranging Equation 13 from the main text as follows, by assuming instantaneous equilibrium at the micropores ($\frac{\partial q_j}{\partial t} = \frac{\mathrm{d}q_j^*}{\mathrm{d}c_j^{\mathrm{m}}}\frac{\partial c_j^{\mathrm{m}}}{\partial t}$).

$$\varepsilon_{\mathrm{p}}\frac{\partial c_j^{\mathrm{m}}}{\partial t} + (1-\varepsilon_{\mathrm{p}})\frac{\mathrm{d}q_j^*}{\mathrm{d}c_j^{\mathrm{m}}}\frac{\partial c_j^{\mathrm{m}}}{\partial t} - \frac{1}{r^2}\frac{\partial}{\partial r}\left(D_{\mathrm{mac}}^{\mathrm{e}} r^2 \frac{\partial c_j^{\mathrm{m}}}{\partial r}\right) = 0 \tag{S8}$$

Here $\frac{\mathrm{d}q_j^*}{\mathrm{d}c_j^{\mathrm{m}}}$ is the local slope of the adsorption isotherm at concentration c_j^{m}. Upon rearranging, this equation can be written in the form

$$\frac{\partial c_j^{\mathrm{m}}}{\partial t} = \frac{1}{r^2}\frac{\partial}{\partial r}\left(\frac{D_{\mathrm{mac}}^{\mathrm{e}}}{\left[\varepsilon_{\mathrm{p}} + \left(1-\varepsilon_{\mathrm{p}}\right)\frac{\mathrm{d}q_j^*}{\mathrm{d}c_j^{\mathrm{m}}}\right]} r^2 \frac{\partial c_i^{\mathrm{m}}}{\partial r}\right) \tag{S9}$$

where the effective pore diffusivity $D_{\mathrm{p}}^{\mathrm{e}}$ is given as follows.

$$D_{\mathrm{p}}^{\mathrm{e}} = \frac{D_{\mathrm{mac}}^{\mathrm{e}}}{\left[\varepsilon_{\mathrm{p}} + \left(1-\varepsilon_{\mathrm{p}}\right)\frac{\mathrm{d}q_j^*}{\mathrm{d}c_j^{\mathrm{m}}}\right]} \tag{S10}$$

For a downward concave isotherm (Type I), the maximum value for the local slope of the isotherm is at the Henry's law region (defined by the dimensionless Henry's law constant K_0) and thus the limiting lower value of effective pore diffusivity $D_{\mathrm{p},0}^{\mathrm{e}}$ will approach the following.

$$D_{\mathrm{p},0}^{\mathrm{e}} = \frac{D_{\mathrm{mac}}^{\mathrm{e}}}{\left[\varepsilon_{\mathrm{p}} + \left(1-\varepsilon_{\mathrm{p}}\right)K_0\right]} \tag{S11}$$

S6 CO_2 equilibrium isotherms and DSL fits

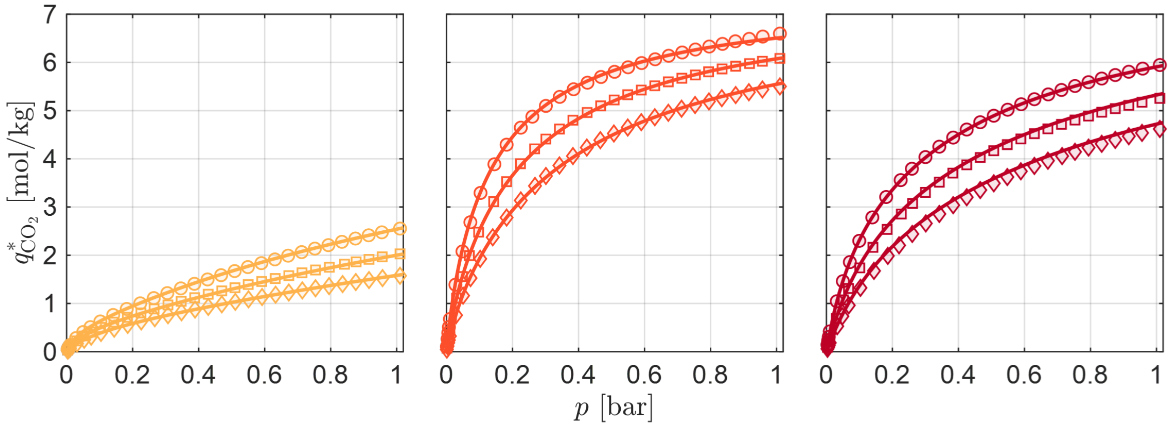

Figure S4: CO_2 adsorption equilibrium isotherms for H-Y (yellow), Na-Y (orange), and NaTMA-Y (maroon) obtained from low pressure volumetric measurements at 288.15 K, 298.15 K and 308.15 K (circles, squares, and diamonds respectively). The solid line through the measured points corresponds to the dual-site Langmuir (DSL) fit of the data given by Equation 4 of the main text.

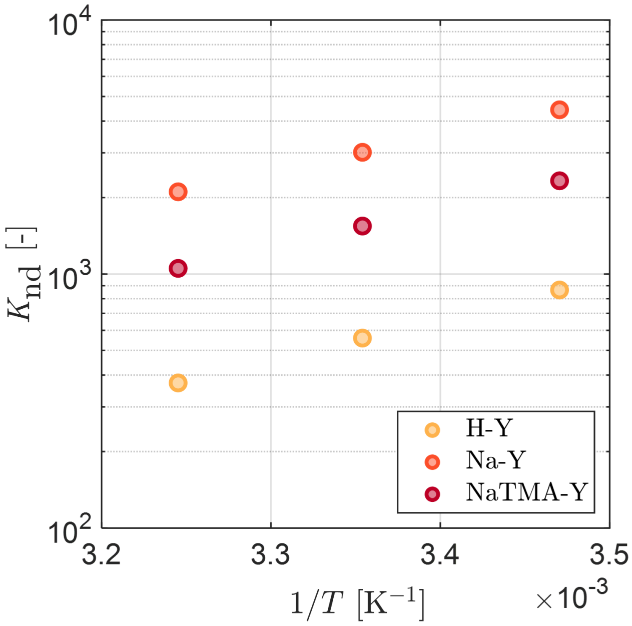

Figure S5: Dimensionless Henry's law constants obtained from the DSL fitted parameters in Table 5 of the main text at 288.15 K, 298.15 K and 308.15 K computed using Equation 6 plotted as a function of the reciprocal of absolute temperature.

S7 Graphical representation of parallel pore model

Figure S6 is a graphical representation of how the parallel pore model is used to compute the theoretical pore diffusivity ($\varepsilon_p D_p$) for a given macropore size distribution. Since the ultimate value for $\varepsilon_p D_p$ is the value of the integral given on the vertical axis at the largest pore width, the model proportionally accounts for contributions from Knudsen and molecular diffusion at every available pore width on the distribution.

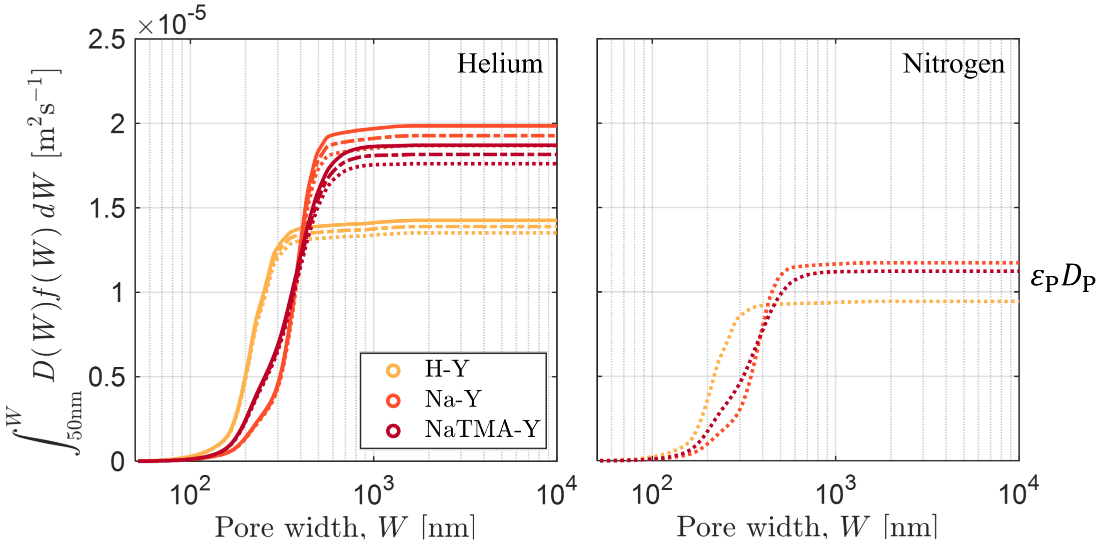

Figure S6: Cumulative plot of the product of the kernel probability distributions for the macropore PSDs ($f(W)$) and the theoretical diffusivity with contributions from Knudsen and molecular diffusivity $D(W)$ (shown in Figure 3) for CO_2 in helium **(left)** and nitrogen **(right)**. As given in Equation 16, the value on the vertical axis on these plots at $W = W_{\text{max}}$ gives the product of the macropore void fraction and theoretical pore diffusivity $\varepsilon_p D_p$ for the given PSD.

References

[1] Azzan, H.; Rajagopalan, A. K.; L'Hermitte, A.; Pini, R.; Petit, C. Simultaneous Estimation of Gas Adsorption Equilibria and Kinetics of Individual Shaped Adsorbents. *Chem. Mater.* **2022**, *34*, 6671–6686.

[2] Rajendran, A.; Kariwala, V.; Farooq, S. Correction procedures for extra-column effects in dynamic column breakthrough experiments. *Chem. Eng. Sci.* **2008**, *63*, 2696–2706.

[3] Joss, L.; Mazzotti, M. Modeling the extra-column volume in a small column setup for bulk gas adsorption. *Adsorption* **2012**, *18*, 381–393.

[4] Friedrich, D.; Mangano, E.; Brandani, S. Automatic estimation of kinetic and isotherm parameters from ZLC experiments. *Chem. Eng. Sci.* **2015**, *126*, 616–624.

[5] Wilkins, N. S.; Rajendran, A.; Farooq, S. Dynamic column breakthrough experiments for measurement of adsorption equilibrium and kinetics. *Adsorption* **2021**, *27*, 397–422.

[6] Rouquerol, J.; Llewellyn, P.; Rouquerol, F. In *Characterization of Porous Solids VII*; Llewellyn, P., Rodriquez-Reinoso, F., Rouqerol, J., Seaton, N., Eds.; Studies in Surface Science and Catalysis; Elsevier, 2007; Vol. 160; pp 49–56.

[7] Hirschfelder, J. O.; Curtiss, C. F.; Bird, R. B. *Molecular theory of gases and liquids*; Wiley: New York, 1954.
